# Action Interpretation Determines the Effects of Go/No-Go and Approach/Avoidance Actions on Food Choice

**DOI:** 10.5334/joc.436

**Published:** 2025-03-05

**Authors:** Zhang Chen, Pieter Van Dessel, Jordi Serverius, Daxun Zhu, Bernd Figner

**Affiliations:** 1 Department of Experimental Psychology, Ghent University, Ghent, Belgium; 2 Department of Experimental Clinical and Health Psychology, Ghent University, Ghent, Belgium; 3 Behavioural Science Institute, Radboud University, Nijmegen, The Netherlands; 4 Donders Institute for Brain, Cognition and Behaviour, Radboud University, Nijmegen, The Netherlands

**Keywords:** go/no-go, approach/avoidance, choice, action execution, action, interpretation, Registered Report

## Abstract

Executing go/no-go and approach/avoidance responses toward objects can increase people’s choices of go over no-go items, and of approach over avoidance items. Some theoretical accounts explain these effects as the results of merely executing these responses (i.e., action execution), while others propose that these choice effects stem from interpreting these motor responses as valenced actions (i.e., action interpretation). To test the role of action execution versus action interpretation in both go/no-go and approach/avoidance responses, we employed a recently developed training that combined both dimensions orthogonally. Participants either pressed a key or not (i.e., go/no-go) to control a shopping cart on screen, to either collect or not collect certain food items (i.e., approach/avoidance). After the training, they repeatedly chose between food items (i.e., candies) for real consumption. When the instructions framed the responses as approach/avoidance actions, participants (*N* = 98) preferred approach items over avoidance items, but did not show preferences between go and no-go items in their choices. In contrast, when the instructions framed the responses as go/no-go actions, participants (*N* = 98) preferred go items over no-go items, but did not show preferences between approach and avoidance items. Despite making the same *actual* responses in both instruction groups, action interpretation determined whether go/no-go or approach/avoidance actions influenced food choice. Disambiguating the interpretation of motor responses as clearly valenced and meaningful actions may therefore be a fruitful way to maximize the effectiveness of response-based behavioral interventions.

## Introduction

Understanding how our preferences for certain objects are formed and can be modified has important implications for explaining and changing human behavior. For instance, people’s preferences for appetitive yet unhealthy foods (e.g., those that contain high sugar, fat, and salt; Breslin, 2013) can lead them to frequently choose and overconsume these foods. Accordingly, changing their preferences for such foods may be one fruitful way to reduce unhealthy eating behavior and the associated negative health consequences.

### Changes in choice induced by go/no-go and approach/avoidance responses

Previous research has shown that executing certain motor responses toward a stimulus can change people’s evaluation of the stimulus (e.g., Van Dessel, Eder, & Hughes, 2018; Veling, Lawrence, et al., 2017). Based on this observation, several computer-based training tasks have been developed as behavior-change tools, such as the go/no-go training (GNG) and the approach/avoidance training (AAT). In GNG, participants consistently respond to some stimuli by pressing a key on the keyboard, whilst not responding to other stimuli by not pressing any key (Veling, Lawrence, et al., 2017). After GNG, people typically choose no-go items less often than go items for consumption (Chen et al., 2019, 2021; Veling et al., 2013a, 2021; Wu et al., 2023). When used as an intervention to reduce unhealthy eating, unhealthy foods can be consistently paired with no-go responses, and healthy foods or non-food items can be consistently paired with go responses. Using such a design, GNG has been shown to reduce choices of unhealthy foods and/or increase choices of healthy foods (Porter et al., 2018; Tzavella et al., 2021; Veling et al., 2013b).

In AAT, people are instructed to repeatedly approach some stimuli and avoid other stimuli. Approach and avoidance actions have been operationalized differently in different versions of AAT. In the joystick version, participants approach a stimulus by pulling a joystick toward themselves (often accompanied by the stimulus becoming larger on screen) and avoid a stimulus by pushing the joystick away from themselves (often accompanied by the stimulus becoming smaller on screen; Rinck & Becker, 2007). In the manikin version, participants approach a stimulus by pressing a key to move a manikin figure on the screen closer to the stimulus, and avoid a stimulus by pressing another key to move the manikin further away from the stimulus (De Houwer et al., 2001). In the eating behavior domain (the behavioral domain that we focus on here), evidence for the effect of AAT on consumption choice is more limited than that for GNG (for recent meta-analyses and systematic reviews, see Aulbach et al., 2019; Yang et al., 2019). Three initial studies failed to find an effect of AAT on food choices immediately following training (Becker et al., 2015). A more recent study used the same procedure from the GNG literature to minimize the methodological differences, and did find that participants overall chose approached food items more often than avoided items for consumption after a joystick AAT training (Veling et al., 2021). This latter finding suggests that AAT can also change consumption choices, and that the reduced efficacy of AAT compared to GNG previously observed in the eating behavior domain may (partly) be explained by the methodological differences in these two lines of research.

### How do go/no-go and approach/avoidance responses influence choices? Action execution versus action interpretation

Executing go/no-go and approach/avoidance responses can change people’s consumption choices. One explanation for these effects is that GNG and AAT change people’s evaluations of trained stimuli. For GNG, people tend to evaluate items that they do not respond to (i.e., no-go items) less positively than items that they respond to (i.e., go items) and items that are not included in the training (i.e., untrained items; e.g., Chen et al., 2016, 2018; Liu et al., 2023; Quandt et al., 2019; for a meta-analysis on this no-go devaluation effect in the eating domain, see Yang et al., 2022). The reduced evaluations of no-go items compared to go items may explain why people tend to choose go items over no-go items for consumption. Similarly, for AAT, several studies have shown that people evaluated approached stimuli more positively than avoided stimuli (Kawakami et al., 2007; Van Dessel et al., 2020; Woud et al., 2013; but see some failed replications, e.g. Van Dessel, De Houwer, Roets, & Gast, 2016; Vandenbosch & De Houwer, 2011). Importantly, effects of GNG and AAT on choice correlated with their effects on evaluation in some studies (Johannes et al., 2021; Veling et al., 2013a, 2021), suggesting that the increased choices of go/approach items over no-go/avoidance items may be driven by changes in stimulus evaluation induced by training.

This in turn raised the question of how go/no-go and approach/avoidance responses may change stimulus evaluation at the psychological process level. Some accounts propose that these effects are the results of merely executing go/no-go or approach/avoidance responses, which we will term *action execution* here. For instance, the Behavior-Stimulus Interaction (BSI) account for GNG (Veling et al., 2008) posits that positive stimuli such as palatable foods trigger an automatic tendency to respond. However, when such stimuli are paired with no-go cues, participants need to inhibit this approach tendency, which results in response conflict. Since response conflicts may be aversive (Dignath et al., 2020; Vermeylen et al., 2020), a stimulus could acquire negative valence after it is paired with the execution of no-go responses. A more recent value-updating account for GNG (Veling et al., 2022) proposes that not responding to highly appetitive items can lead to prediction errors, which in turn reduces the evaluation of appetitive stimuli. This account thus ascribes the no-go devaluation effect to prediction errors, rather than response conflicts. In contrast, the devaluation-by-inhibition account for GNG proposes that no-go responses are associated with punishment while go responses are associated with reward (Guitart-Masip et al., 2014; Verbruggen et al., 2014). This idea is conceptually similar to the motivational systems account for AAT (Neumann et al., 2003), which posits that positive valence is linked to the approach motivational system and negative valence is linked to the avoidance motivational system. According to these two accounts, executing no-go or avoidance actions activates negative valence, while executing go or approach actions activates positive valence, which changes stimulus evaluations. Although the underlying mechanisms proposed by these accounts differ, they all share the core idea that merely executing go/no-go and approach/avoidance actions is sufficient to change stimulus evaluation.

Another explanation for why go/no-go and approach/avoidance responses can change stimulus evaluation is because they are often interpreted as valenced actions (which we will term *action interpretation* here). For instance, the common coding account (Eder & Klauer, 2009; Eder & Rothermund, 2008) for AAT proposes that motor responses acquire valence because they are interpreted as valenced actions based on task instructions and task goals. For instance, approach and avoidance actions are often described by verbal labels as *toward* and *away* from oneself, which have evaluative meaning. This valence then becomes a part of the representation of an action, and is co-activated when the action is executed, thereby influencing the evaluation of a stimulus. The inferential account (Van Dessel, Hughes, & De Houwer, 2018b), which was also primarily developed to explain AAT effects, proposes that actions change stimulus evaluation because participants make cognitive inferences based on their actions. Approach may be interpreted as a positively valenced action (e.g., as selecting an item) and avoidance may be interpreted as a negatively valenced action (e.g., as rejecting an item). This may lead people to infer that they like approached items and dislike avoided items, leading to inferences that promote changes in stimulus evaluation. Again, the exact mechanisms proposed by these two accounts differ, but they all share the core assumption that motor responses acquire evaluative meanings because they are *interpreted* as valenced actions, rather than that the mere execution of these responses is sufficient to change stimulus evaluation.

Some studies have examined action interpretation in both GNG and AAT, and the results corroborated the crucial role of action interpretation in determining the GNG and AAT effects. For instance, Laham et al. (2014) showed that the joystick AAT led to changes in implicit evaluation when approach action (i.e., pulling a joystick toward oneself) was framed as *collecting* something and avoidance action (i.e., pushing a joystick away from oneself) was framed as *discarding* something, but not when such action framing was not used. Collecting and discarding something have clear evaluative connotations, which explains why joystick movements can change stimulus evaluation when they are interpreted as these valenced actions. Other studies found that, in the manikin AAT, participants evaluated approached items more positively than avoided items when their actions were described as approaching and avoiding an item (i.e., decreasing and increasing the distance between the manikin and an item; Van Dessel, Eder, & Hughes, 2018). However, when the same responses were described as moving the manikin *upward* or *downward* on the screen, participants reported increased liking for *upward* items than *downward* items. No effect of distance change itself was observed on stimulus evaluation (Van Dessel, Eder, & Hughes, 2018). Lastly, in a recent study on GNG by Houben (2023), the meaning of go and no-go actions were manipulated via instructions. For one group of participants, go action was framed as *taking* something and no-go action was framed as *not taking* something, while for the other group, go was framed as *throwing away* something and no-go was framed as *keeping* something. Explicit evaluations of chocolate stimuli were lower after they had been paired with negatively framed actions (i.e., not taking and throwing away) compared to positively framed actions (i.e., taking and keeping), regardless of whether participants executed go or no-go responses. No clear effect was observed for fruit stimuli though, a second type of foods used in the training (Houben, 2023). Together, these results suggest that the same motor responses can be interpreted differently depending on task instructions and that action interpretation, rather than the executed motor responses per se, determines the effects of GNG and AAT on stimulus evaluation.

### Combining go/no-go and approach/avoidance actions

In addition to underscoring the role of action interpretation, the results reviewed above further suggest that there may be important commonalities in how go/no-go and approach/avoidance actions change stimulus evaluation. However, most research on GNG and AAT have been conducted in isolation from each other. As a result, different theoretical accounts have been proposed for GNG and AAT separately (see the brief overview above), with little cross-talk between these two strands of research. However, GNG and AAT share many similarities. Both tasks involve the repeated execution (or withholding) of simple motor responses toward certain objects. Furthermore, the theoretical accounts for GNG and AAT show much conceptual overlap (e.g., the devaluation-by-inhibition account for GNG and the motivational systems account for AAT), and can be easily translated from one line of research to the other. For instance, the BSI theory for GNG attributes the no-go devaluation effect to response conflicts when people inhibit their tendency to respond to appetitive stimuli (Veling et al., 2008). It is conceivable that avoiding a positive stimulus and approaching a negative stimulus may similarly trigger response conflicts, which may impact stimulus evaluation (e.g., Centerbar & Clore, 2006). Similarly, while the common coding account (Eder & Klauer, 2009; Eder & Rothermund, 2008) and the inferential account (Van Dessel, Hughes, & De Houwer, 2018b) are developed primarily based on research on AAT, the recent findings by Houben (2023) show that action interpretation similarly plays a crucial role in GNG, in line with the theoretical propositions of these two accounts. GNG and AAT may therefore share important commonalities in their underlying mechanisms. More cross-pollination between these two lines of research can allow us to develop more comprehensive theories of how actions impact stimulus evaluation and subsequent consumption behavior (Houben & Aulbach, 2023).

Direct comparisons between GNG and AAT will benefit from using comparable research protocols (Houben & Aulbach, 2023; Veling et al., 2021). To this end, Chen and Van Dessel (2024) recently developed a novel training task that combined go/no-go and approach/avoidance actions in an orthogonal manner. In this task, participants either pressed a key or not (i.e., go/no-go actions) to control the location of a virtual shopping cart on the screen. Orthogonal to participant’s go/no-go actions, food items fell either inside or outside the shopping cart, as operationalizations of approach and avoidance consequences (similar to the manikin AAT). Two groups of participants received the training, with the task instructions manipulated between groups. When the task instructions and cues indicated to participants whether they needed to respond or not to certain items (i.e., go/no-go actions), a typical no-go devaluation effect was observed. Participants evaluated no-go items less positively than both go and untrained items. No effect of approach/avoidance actions on stimulus evaluation was observed. However, when the task instructions and cues indicated to participants whether they needed to have certain items either inside or outside the shopping cart (i.e., approach/avoidance actions), an AAT effect was observed. People evaluated approached items more positively than untrained items, and avoided items less positively than untrained items. No effect of go/no-go actions on evaluation was observed. These findings cannot be easily explained by the accounts that emphasize mere action execution, since in both conditions participants made the same motor responses, yet the effects of the training differed depending on task instructions. Instead, these findings further support the idea that action interpretation determines the effects of go/no-go and approach/avoidance actions on stimulus evaluation. The same responses can be interpreted as valenced actions along one of two orthogonal dimensions, and the effect of training on stimulus evaluation aligned with action interpretation. From this perspective, the findings by Chen and Van Dessel (2024) can be seen as a conceptual replication of previous work (Houben, 2023; Laham et al., 2014; Van Dessel, Eder, & Hughes, 2018).

### The present research

All studies that have examined action interpretation in GNG and AAT have focused on stimulus evaluation (Chen & Van Dessel, 2024; Houben, 2023; Laham et al., 2014; Van Dessel, Eder, & Hughes, 2018). It is therefore unclear whether action interpretation may similarly influence other behavioral effects of GNG and AAT, such as people’s consumption choices. Training-induced changes in stimulus evaluation may mediate the effects of GNG and AAT on consumption choices, or the effects of GNG and AAT on both stimulus evaluation and consumption choice may be underpinned by the same mental processes. In both cases, action *interpretation* should similarly determine the effects of GNG and AAT on stimulus evaluation and consumption choice. However, although stimulus evaluation undoubtedly plays an important role in people’s choices, choices are also shaped by other processes. For instance, rapidly executing go responses may increase attention toward go items (Itzkovitch et al., 2022; Schonberg & Katz, 2020; Schonberg et al., 2014), while inhibiting responses may reduce attention for concurrent no-go items (Chiu & Egner, 2015a; Chiu & Egner, 2015b). Selective attention toward one object over another can change people’s choices while these objects are presented simultaneously (Armel et al., 2008), which can be an alternative explanation for how go/no-go actions influence choices. Importantly, the attentional influences of go/no-go actions presumably do not depend on how these actions are interpreted, which leads to the possibility that the effect of GNG on choices may not depend on action interpretation.

Examining this possibility also has important practical implications. There is a growing interest in using GNG and AAT as behavior-change tools in applied settings. For instance, in the eating behavior domain, some studies have shown that repeated training with GNG can facilitate weight loss (Allom & Mullan, 2015; Forman et al., 2019; Lawrence et al., 2015; Stice et al., 2017; Veling et al., 2014), but other studies have failed to find this effect (Adams et al., 2021; Allom & Mullan, 2015; Carbine et al., 2021; Memarian et al., 2021; Najberg et al., 2021; Yang et al., 2021). Disambiguating the evaluative meanings of go/no-go and approach/avoidance actions may increase training efficacy (e.g., Van Dessel, Hughes, & De Houwer, 2018a). However, before such changes are implemented for applied use, it is important to examine action interpretation beyond stimulus evaluation. People make numerous dietary choices everyday, with large cumulative health consequences in the long run. Examining action interpretation in the effects of GNG and AAT on consumption choices will therefore provide important insights into whether disambiguating action interpretation can effectively increase training efficacy in applied settings.

In the present research, we examined the role of action interpretation in changes in choice induced by go/no-go and approach/avoidance actions, using the same task by Chen and Van Dessel (2024). Participants either responded or did not respond (i.e., go/no-go) to control a shopping cart on screen, with food items falling either inside or outside the cart as a result (i.e., approach/avoidance). For half of them, the cues indicated whether they should make go or no-go actions, while for the other half, the cues indicated whether they should make approach or avoidance actions. After the training, all participants received a food choice task, in which they repeatedly chose between food items for consumption (Chen et al., 2019). Using this setup, we examined whether the same responses would lead to different effects on consumption choices, depending on how the responses were interpreted.

## Method

### Ethics, transparency and openness

The current research was conducted according to the ethical rules presented in the General Ethical Protocol of the Faculty of Psychology and Educational Sciences of Ghent University. All participants provided written informed consent. The current manuscript achieved level 6 of bias control according to the policies of Peer Community in Registered Reports: *No part of the data or evidence that will be used to answer the research question yet exists and no part will be generated until after IPA*. All experimental materials, raw data and analysis code are available at https://osf.io/24apk/. The Stage 1 manuscript received in-principle acceptance on September 30th, 2024 (https://osf.io/bn5xa). Both the Stage 1 and Stage 2 versions of the current manuscript have been positively recommended by PCI RR (see https://rr.peercommunityin.org/articles/rec?id=846 and https://doi.org/10.24072/pci.rr.100951) for the recommendations).

### Sample size

The recent study by Chen and Van Dessel (2024) combining GNG and AAT found an effect size of Cohen’s *d* of 0.563 for go/no-go actions on stimulus evaluation (when the cues indicated go/no-go actions), and Cohen’s *d* of 0.833 for approach/avoidance actions on evaluation (when the cues indicated approach/avoidance actions). When a food choice task was used, previous work has found an effect size of Cohen’s *d* of 0.533 for GNG on food choices (Chen & Veling, 2022),^1^ and Cohen’s *d* of 0.343 for AAT on food choices (Veling et al., 2021). The effect size of GNG on stimulus evaluation in Chen and Van Dessel (2024) is thus comparable to the effect of GNG on choices, while the effect of AAT on stimulus evaluation in Chen and Van Dessel (2024) is numerically larger than that on choices in Veling et al. (2021). Note that Veling et al. (2021) used the joystick version of AAT, while Chen and Van Dessel (2024) based their task on the manikin version of AAT. Furthermore, approach action in Chen and Van Dessel (2024) was operationalized as foods falling into one’s shopping cart, and avoidance as foods falling outside one’s shopping cart. These action consequences likely provide clearer evaluative meanings than those used in the joystick AAT (i.e., food items becoming larger or smaller on the screen). These differences may explain why approach/avoidance actions had a larger effect on stimulus evaluation in Chen and Van Dessel (2024) than on choices in Veling et al. (2021). Since we used the training task from Chen and Van Dessel (2024), we expected the AAT effect on food choices to be at least as large as that of GNG here. We therefore expected the effect size for both the effects of go/no-go and approach/avoidance actions on choices to be around Cohen’s *d* of 0.533. Given the inherent uncertainty in effect size estimates, we used Cohen’s *d* of 0.426 (i.e., 0.533 * 80%) as the expected effect size in a power analysis in G*Power (version 3.1.9.6; Faul et al., 2007), which showed that 60 participants were needed (with a two-sided one sample *t* test) for 90% power with an alpha level of .05.

Chen and Van Dessel (2024) further showed that go/no-go actions had a larger effect on evaluation when the cues indicated go/no-go actions in comparison to when the cues did not indicate go/no-go actions, Hedge’s *g_av_* = 0.611. Likewise, approach/avoidance actions had a larger effect on evaluation when the cues indicated this dimension than when the cues did not indicate this dimension, Hedge’s *g_av_* = 0.923. Again, to be conservative, we used Cohen’s *d* of 0.488 (i.e., 0.611 * 80%) in a power analysis, which showed that 90 participants per condition (180 participants in total) are needed (with a two-sided independent samples *t* test) for 90% power with an alpha level of .05.

Note that we used *t* tests in the power analysis above, while we planned to use Bayesian mixed-effects models to analyze the data (see below). The power analyses were therefore meant to provide ballpark estimates for the sample sizes needed. To be more conservative in our sample size planning, we therefore used 80% of the expected effect size and 90% power in the power analyses above. To allow for potential exclusions, we decided to recruit 100 participants per condition (*N* = 200 in total). In case the sample size in an instruction group is below 90 after exclusions (see below), we will continue recruiting participants for that specific instruction group, until the final sample sizes in both groups are 90 after exclusions.

### Participants

Participants were recruited via the SONA participation system at Ghent University and received one course credit for participation. Participants needed to be at least 18 years old, which was the minimal age to provide informed consent. However, based on our past experience, some participants might be younger than 18 years old, but also needed course credits for their education program. We decided to allow these participants to participate in case they had already signed up for the experiment, but immediately delete their data after completion. Note that the planned initial sample size of 200 did not include these participants. In other words, we planned to initially recruit 200 participants who were at least 18 years old.

Two hundred participants took part in the experiment in October, 2024. Another 9 participants were younger than 18 years old, and their data were deleted immediately after testing, as planned. Another 3 participants did not give consent to (re)use their data. We did not anticipate this, and thus did not pre-register this as an exclusion criterion. However, since these participants needed credits for their education, we decided to let them participate, but deleted their data immediately after completion.

Four participants met the pre-registered exclusion criteria (see below). The final sample thus consisted of 196 participants, with 98 participants in each instruction group (the go/no-go instruction group, *M_age_* = 18.47, *SD_age_* = 1.26, 9 men, 89 women, *M_BMI_* = 21.99, *SD_BMI_* = 3.41, *M_hunger_* = 6.94, *SD_hunger_* = 1.65, *M_restraint_* = 13.17, *SD_restraint_* = 5.20; the approach/avoidance instruction group, *M_age_* = 18.41, *SD_age_* = 1.21, 11 men, 87 women, *M_BMI_* = 21.75, *SD_BMI_* = 3.01, *M_hunger_* = 6.94, *SD_BMI_* = 1.73, *M_restraint_* = 12.86, *SD_restraint_* = 5.12).

### Apparatus and materials

The experiment was programmed in jsPsych (version 7.2.1; de Leeuw, 2015). Sixty images of different candies created by Chen et al. (2019) were used. Note that the original images showed the candies on a plate. For the current experiment, we slightly modified the images by removing the plate and making the background transparent. This was done to enhance the visual feedback of candies falling into the shopping cart in the training.

### Procedure

Participants signed up for the experiment via the participation system. The eligibility criteria were: (1) being at least 18 years old (but see above), (2) being able to consume candies (e.g., no food allergies), and (3) not having participated in previous studies using the same task. In line with previous experimental protocols (e.g., Chen et al., 2019), they were asked to not eat anything for at least 3 hours before the start of the experiment. As such, testing only started at 11.00 am or later.

Participants were tested in small groups, with up to 6 people per session. Upon arrival, they were shown a selected collection of candies, and told that in the experiment they would eat some candies depending on their choices. They were then asked to inspect the candies. This was done to ensure that (1) participants understood that the choices that they were going to make were real, and (2) they could eat candies (e.g., no food allergies). They were then seated in front of computers individually, and asked to read and sign an informed consent. The experiment then commenced.

#### Pre-training rating

**Figure 1:**
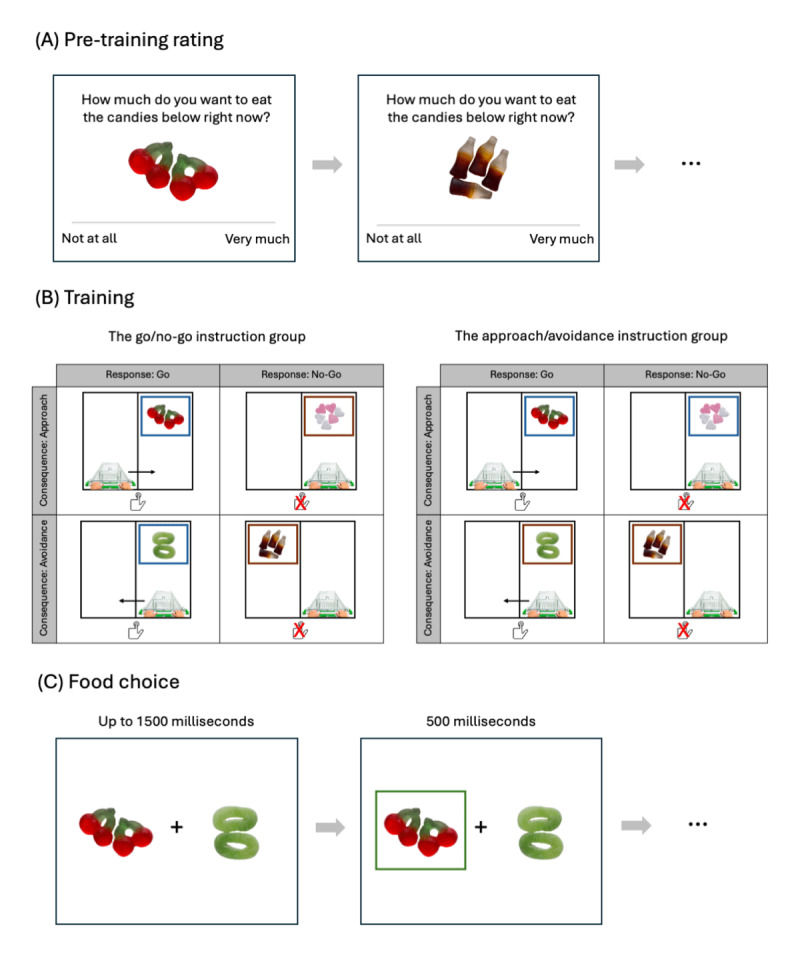
Schematics of the main tasks. **(A)** Pre-training rating. **(B)** Training. Note that the colored frames (blue and brown) indicate go/no-go conditions in the go/no-go instruction group, and indicate approach/avoidance conditions in the approach/avoidance instruction group. The solid arrows in the Go-Approach and Go-Avoidance conditions indicate how the cart moves after participants respond, and are not shown in the task. **(C)** Food choice. The candy images are from Chen et al. (2019). The shopping cart image is from pngwing.com, and the hand pushing button image is from stockio.com.

Participants first reported their age and gender (with four options, *man, woman, non-binary* and *I do not want to say*). They then received a rating task, in which each of the sixty candy images was presented one by one. For each image, they were asked the question “how much do you want to eat the candies below right now?”. Participants answered with a 200-point slider (–100 = *Not at all*, 100 = *Very much*; see Figure 1, panel A). Participants could click anywhere on the slider and a cursor would then appear on the clicked location. They could further adjust the position of the cursor until it accurately reflected their rating. They could then click on a ‘Continue’ button beneath the slider to advance to the next trial. The rating task was self-paced, without time limits.

#### Item ranking and selection

**Figure 2:**
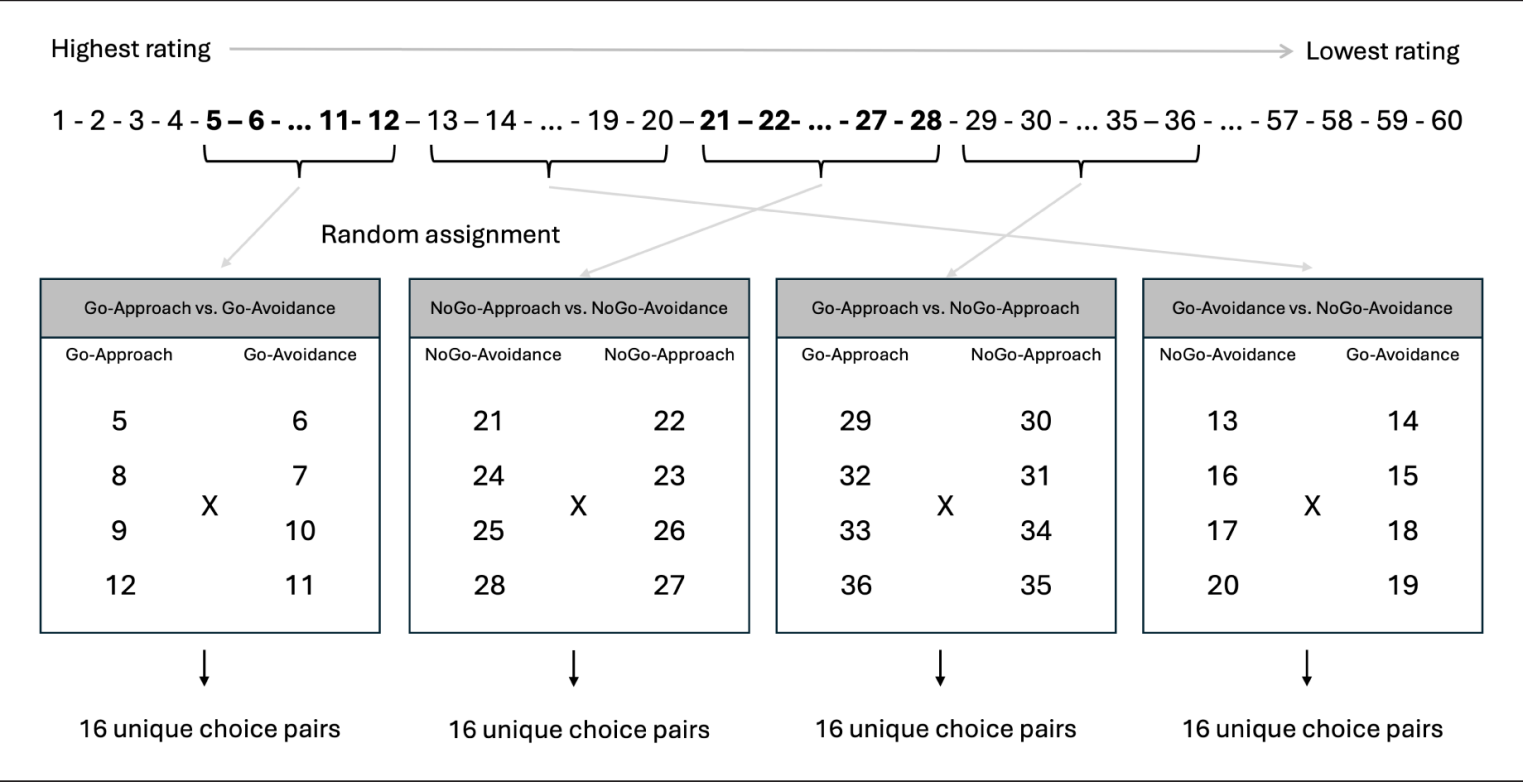
An illustration of the item selection procedure.

The program then ranked all candy images from the highest rating till the lowest, for each participant individually (Figure 2). Ties were broken randomly. In the food choice task after the training, we created four types of experimental trials: Go-Approach vs. Go-Avoidance, NoGo-Approach vs. NoGo-Avoidance (these were used to assess the effects of approach vs. avoidance actions), Go-Approach vs. NoGo-Approach, Go-Avoidance vs. NoGo-Avoidance (these were used to assess the effects of go vs. no-go actions). We thus selected and assigned candy images into one of these four types of choice trials. More concretely, items ranked from 5 till 36 were used, with every 8 items as one set (i.e., 5–12, 13–20, 21–28 and 29–36, respectively). In total, 32 items were selected. Note that we selected items with relatively high ratings, since we were mainly interested in how the training would impact choices for relatively appetitive items. These four sets were randomly assigned into the four types of choice trials as described above. Within each set, the 8 items were assigned into one of the two corresponding training conditions randomly, in a counterbalanced manner. For instance, in the example in Figure 2, the set 5–12 was randomly assigned to the Go-Approach vs. Go-Avoidance condition. Within this set, items ranked 5, 8, 9, 12 was further randomly assigned into the Go-Approach condition, and items ranked 6, 7, 10, 11 was assigned into the Go-Avoidance condition (but it could also be the other way around). This selection procedure has been used in multiple previous studies (e.g., Schonberg et al., 2014; Veling, Chen, et al., 2017), to match the average ratings of two conditions. In the example above, before the training, participants should have no preference between Go-Approach and Go-Avoidance items based on their ratings. Any preference for one condition over the other in participants’ choices could thus be attributed to the training.

#### Training

The selected images were then used in the same training task developed by Chen and Van Dessel (2024). Participants were assigned into either the go/no-go or the approach/avoidance instruction condition, depending on the anonymous subject ID that they received upon arrival in the laboratory (odd number = the go/no-go instruction group, even number = the approach/avoidance instruction group). Participants were told that they were a customer in a virtual candy store, and the shopping cart on screen belonged to them. The candy store had two lanes, left and right. The shopping cart was placed at the bottom of one of the lanes. At the beginning of each trial, a candy image appeared near the top of a lane, and gradually moved from top to bottom, as if participants were pushing the cart forward and getting closer to the candies. 300 milliseconds after image onset, a colored frame appeared around the image (blue or brown). Participants in the go/no-go instruction condition were told that the cues indicated whether they needed to respond or not (Figure 1, panel B, left). If the color was assigned to the go condition, participants needed to press the space bar once as quickly as possible. If the color was assigned to the no-go condition, participants did not need to press any key. The assignment of the two colors into the go and no-go conditions was counterbalanced across participants. Participants were further told that they could press the space bar at most once within a trial, and each time when they pressed the space bar, the shopping cart would switch from one lane to the other. In both the go and no-go conditions, the image disappeared after nearly reaching the bottom of the lane, creating the visual impression of candies falling either inside or outside the shopping cart. One trial lasted for 2 seconds, with inter-trial intervals randomly varying between 1 and 1.5 seconds (in steps of 100 milliseconds).

The training was the same for participants in the approach/avoidance instruction condition, except that the cues indicated whether they should let the candies fall inside (i.e., approach) or outside (i.e., avoid) the shopping cart (Figure 1, panel B, right). The assignment of colors into approach/avoidance conditions was counterbalanced across participants.

Items in the Go-Approach condition always appeared on a different lane than the shopping cart. Participants had to press the space bar (i.e., go), after which the candies eventually fell into the shopping cart (i.e., approach). Items in the Go-Avoidance condition always appeared on the same lane as the shopping cart. After participants responded (i.e., go), the items eventually fell outside the shopping cart (i.e., avoidance). In contrast, for items in the NoGo-Approach and NoGo-Avoidance conditions, participants did not respond (i.e., no-go), and the items fell either inside (i.e., NoGo-Approach) or outside the cart (i.e., NoGo-Avoidance) as a result. Note that participants in the two instruction conditions made the same motor responses, with the only difference being whether the cues indicated the go/no-go or the approach/avoidance dimension, to manipulate action interpretation.

Participants first received a practice block, with 4 images in each training condition. These images (ranked 41–56) were used during practice only. Each image was randomly presented once, resulting in 16 trials for one practice block. After the practice block, participants received feedback on their accuracy. In case their accuracy was below 75%, they were asked to read the task instruction again, and received a new practice block. They could practice up to 3 times, after which they were allowed to proceed to the experimental blocks, even if their accuracy in the last practice block was still below 75%.

For the experimental blocks, each of the 32 selected images was randomly presented once in each block. After every two blocks, participants received feedback on their accuracy in the preceding two blocks, and could take a short break if necessary. The whole training consisted of 14 blocks, resulting in 448 trials in total.

#### Food choice task

Participants then received a food choice task (Figure 1, panel C). They were told that they would make a series of binary food choices. At the end of the experiment, one trial would be randomly selected, and they would receive a small bag of the candies that they had chosen on the selected trial. Two images of candies were presented side by side. Participants chose which of the two candies they would like to eat, by pressing the U key for the left candies and the I key for the right candies. They were asked to make their choices within 1500 milliseconds. If they chose in time, a green frame was presented for 500 milliseconds around the chosen candies as confirmation. If they did not choose in time, a text message “Too late!” was presented for 1000 milliseconds, and the choice pair was presented again at the end of the block, until a choice was registered. The inter-trial intervals randomly varied between 1 and 1.5 seconds, in steps of 100 milliseconds.

Participants first received a practice block of 8 choice trials, with items that were not used in the experimental blocks of the training (ranked 41–56). The experimental part of the choice task consisted of two types of trials, experimental trials and filler trials. For the experimental trials, we used items that were used in the training to construct choice pairs. As mentioned above in *Item ranking and selection*, we had four types of choice trials, namely Go-Approach vs. Go-Avoidance, NoGo-Approach vs. NoGo-Avoidance, Go-Approach vs. NoGo-Approach and Go-Avoidance vs. NoGo-Avoidance. For each trial type, each item from one of the two conditions was paired once with each item from the other condition, resulting in 16 unique choice pairs (Figure 2). For instance, for the Go-Approach vs. Go-Avoidance condition, each of the four Go-Approach items was paired once with each of the Go-Avoidance items. In total, 64 (i.e., 16 per condition, multiplied by 4) unique choice pairs were constructed. In addition to these experimental choice trials, we also included some filler trials. For these filler trials, 4 items with high ratings (ranked 1–4) were each paired once with 4 items with relatively low ratings (ranked 37–40), resulting in 16 unique choice pairs. Each of the 80 unique choice pairs (64 experimental and 16 filler trials) was randomly presented once in one block. The choice task consisted of two blocks (160 trials), to counterbalance the left versus right location of the items within each pair. After the first block, participants could take a short break.

At the end of the second block, we added 3 choice trials with 6 types of candies that were present in the laboratory. Unbeknownst to the participants, the program always randomly picked from these three trials to determine which candies they would receive. We implemented this to limit the amount of candies that we had to purchase, to reduce food waste (see e.g. Chen et al., 2019).

#### Memory tasks

After the food choice task, participants received two memory tasks, in a counterbalanced order. In the go/no-go memory task, participants were shown each of the 32 selected images randomly one by one, and asked to report for each item whether they made go or no-go responses. The five answer options were ‘sure did not press’, ‘maybe did not press’, ‘do not remember’, ‘maybe did not press’, ‘sure did not press’. In the approach/avoidance memory task, participants were similarly shown each of the 32 selected images randomly one by one, and asked to report for each item whether it eventually fell inside or outside their shopping cart. The five answer options were ‘sure outside’, ‘maybe outside’, ‘do not remember’, ‘maybe inside’, ‘sure inside’. The memory tasks were self-paced without time limits. No feedback was provided. Previous work has shown that memory of stimulus-response contingencies correlated with training effects in both GNG and AAT (e.g., Chen & Veling, 2022; Van Dessel, De Houwer, & Gast, 2016). We therefore included memory tasks to potentially explore the role of memory in our novel training paradigm.

#### Post-training rating

As an exploratory measure, the same rating task was administered again, to explore whether stimulus evaluation had changed compared to before the training.

#### Questionnaires

At the end of the experiment, participants filled out the 10-item restrained eating scale (Herman & Polivy, 1980), reported their height (in centimeters), weight (in kilograms), how hungry they were (using a 9-point Likert scale, with 1 = *Not at all*, 9 = *Very much*), and when they had their last meal (‘Less than 1 hour ago’, ‘1–3 hours ago’, ‘3–5 hours ago’, ‘More than 5 hours ago’). These measures were included to provide descriptive information about the sample regarding their eating behavior. They also offer additional context that may be relevant in exploratory analyses to understand how these factors might influence the effectiveness of the training paradigm. One trial from the choice task (from only the last three trials) was then randomly selected and revealed, and participants received a small bag of the candies that they had chosen on the selected trial. They were then debriefed, thanked, and received one course credit as compensation.

### Data analysis

Data were analyzed using R (version 4.2.1; R Core Team, 2022), with the following R packages: afex (version 1.2.0; Singmann et al., 2022), bayesplot (version 1.10.0; Gabry & Mahr, 2022), bayestestR (version 0.13.0; Makowski et al., 2022), brms (version 2.18.0; Bürkner, 2022), cmdstanr (version 0.5.3; Gabry & Češnovar, 2022), ggpubr (version 0.6.0; Kassambara, 2023), kableExtra (version 1.3.4; Zhu, 2021), knitr (version 1.41; Xie, 2022), loo (version 2.5.1; Vehtari et al., 2022), MASS (version 7.3.58.1; Ripley, 2022), Rmisc (version 1.5.1; Hope, 2022), sjPlot (version 2.8.12; Lüdecke, 2022), tidybayes (version 3.0.2; Kay, 2022), and tidyverse (version 1.3.2; Wickham, 2022). We also used JASP (version 0.19.1; JASP Team, 2024) to conduct Bayesian ANOVAs.

#### Data exclusion

Participants who met one of the following two criteria were excluded from further analysis: (1) restarting the experiment after having completed some trials (0 participant), and (2) having an accuracy 3 standard deviations below the sample mean in their instruction condition, and below 90% in any of the four conditions in the training (4 participants). Note that Chen and Van Dessel (2024) had an extra exclusion criterion based on missing data (due to online testing), which was not a concern here as the current experiment was conducted in the laboratory. The remaining two exclusion criteria from above were the same as in Chen and Van Dessel (2024), to make this follow-up experiment as comparable as possible with the previous study.

#### Pre-registered analyses

##### Ratings before the training

For each participant, we first selected items that were assigned into the Go-Approach vs. Go-Avoidance and the NoGo-Approach vs. NoGo-Avoidance choice trials (e.g., the two cells on the left in Figure 2). These trials were used to assess the effects of approach/avoidance actions on choice, while holding go and no-go actions constant. The average ratings of the items in the four training conditions were then computed, and submitted to a 2 (response, go vs. no-go; within-subjects) by 2 (consequence, approach vs. avoidance; within-subjects) by 2 (instruction group, go/no-go vs. approach/avoidance; between-subjects) Bayesian repeated-measures ANOVA in JASP. We used the default prior settings in JASP, and computed the Bayes factor for each effect across matched models. Bayes factors (*BF*_01_) quantified the relative likelihood of the data under the null hypothesis against that under the alternative hypothesis. We expected the *BF*_01_ for the main effect of consequence, and that for the interaction effect between consequence and instruction group to be larger than 3, which would provide support for the null hypothesis (Wagenmakers et al., 2018). This would suggest that before the training, the average ratings for the approach and avoidance items were matched.

Similarly, the items assigned into the Go-Approach vs. NoGo-Approach and the Go-Avoidance vs. NoGo-Avoidance choice trials (e.g., the two cells on the right in Figure 2) were selected. These trials were used to assess the effects of go/no-go actions on choice, while holding approach and avoidance actions constant. The average ratings of the items in the four training conditions were computed, and submitted to a 2 (response, go vs. no-go; within-subjects) by 2 (consequence, approach vs. avoidance; within-subjects) by 2 (instruction group, go/no-go vs. approach/avoidance; between-subjects) Bayesian repeated-measures ANOVA. We expected the *BF*_01_ for the main effect of go/no-go response, and that for the interaction between response and instruction group to be larger than 3. This would suggest that before the training, the average ratings for the go and no-go items were matched.

##### Choices on the filler trials

In the choice task, we included filler trials in which participants chose between an item with a high rating (ranked from 1 till 4) and an item with a relatively low rating (ranked from 37 till 40). Choices on these filler trials were analyzed with a hierarchical logistic regression in *brms*, with whether participants chose the item with a higher rating or not as the dependent variable. The instruction group was included as a between-subjects predictor (go/no-go = 0.5, approach/avoidance = –0.5). We included the maximum random structure (Barr et al., 2013), by using participant, the candy presented on the left, and the candy presented on the right as three grouping variables. Random intercept per participant, and random intercept and random slope for instruction group per candy were included. The pseudocode for the brms model was: choice ∼ instruction group + (1 ∣ participant) + (instruction group ∣ left candy) + (instruction group ∣ right candy). We expected the 95% credible interval of the intercept to be larger than 0 (i.e., excluding 0), indicating that overall participants in both groups selected highly-rated items more frequently than lowly-rated items.

##### Choices on the experimental trials

The main analysis focused on the choices on the experimental trials. We conducted two separate analyses, (1) one for the Go-Approach vs. Go-Avoidance and NoGo-Approach vs. NoGo-Avoidance trials, and (2) one for the Go-Approach vs. NoGo-Approach and the Go-Avoidance vs. NoGo-Avoidance trials. For the first analysis, we used whether participants chose the Approach or the Avoidance item on each trial as the dependent variable (Approach = 1, Avoidance = 0). Whether both items were Go or NoGo (i.e., response) was used a within-subjects predictor, and the instruction group was used as a between-subjects predictor. We used effect coding for the predictors (Go = 0.5, NoGo = –0.5; approach/avoidance instruction group = 0.5, go/no-go instruction group = –0.5). Again, the maximum random structure was used. The pseudocode for the brms model was: choice ∼ response * instruction group + (response ∣ participant) + (response * instruction group ∣ left candy) + (response * instruction group ∣ right candy). We expected a statistically credible effect for the instruction group, such that participants in the approach/avoidance instruction group would select Approach items more often than those in the go/no-go instruction group. Furthermore, we expected the approach/avoidance instruction group to overall choose Approach items more than 50% of the time. In other words, the 95% CI for the estimated intercept for the approach/avoidance instruction group from the model above was expected to be credibly larger than 0.

For the second analysis, we used whether participants chose the Go or the NoGo item on each trial as the dependent variable (Go = 1, NoGo = 0). Whether both items were associated with Approach or Avoidance (i.e., consequence) was used a within-subjects predictor, and the instruction group was used as a between-subjects predictor. We used effect coding for the predictors (Approach = 0.5, Avoidance = –0.5; go/no-go instruction group = 0.5, approach/avoidance instruction group = –0.5). The pseudocode for the brms model was: choice ∼ consequence * instruction group + (consequence ∣ participant) + (consequence * instruction group ∣ left candy) + (consequence * instruction group ∣ right candy). We expected a statistically credible effect for the instruction group, such that participants in the go/no-go instruction group would select Go items more often than the approach/avoidance instruction group. Furthermore, we expected the go/no-go instruction group to overall choose Go items more than 50% of the time. That is, the 95% CI for the estimated intercept for the go/no-go instruction group from the model above was expected to be credibly larger than 0. See Table A1 in the Appendix for the study design table.

#### Exploratory analyses

We conducted exploratory analyses on participants’ performance in the training. For each participant, we computed their accuracy in the experimental blocks in each of the four training conditions. Accuracy data were then analyzed with a 2 (response, go vs. no-go; within-subjects) by 2 (consequence, approach vs. avoidance; within-subjects) by 2 (instruction group, go/no-go vs. approach/avoidance; between-subjects) Bayesian repeated-measures ANOVA in JASP. We further computed the mean response times on the correct Go-Approach and Go-Avoidance trials. The mean go response times were analyzed with a 2 (consequence, approach vs. avoidance; within-subjects) by 2 (instruction group, go/no-go vs. approach/avoidance; between-subjects) Bayesian repeated-measures ANOVA.

Furthermore, we examined participants’ performance in the memory tasks, as a kind of “manipulation check,” since the approach/avoidance instruction group would be expected to remember the approach/avoidance contingencies better than the go/no-go instruction group, whereas the go/no-go instruction group would be expected to remember the go/no-go contingencies better than the approach/avoidance group. To accomplish this, we computed the average responses in each cell in the approach/avoidance and go/no-go memory tasks separately, and analyzed them with 2 (response, go vs. no-go; within-subjects) by 2 (consequence, approach vs. avoidance; within-subjects) by 2 (instruction group, go/no-go vs. approach/avoidance; between-subjects) Bayesian repeated-measures ANOVAs. Furthermore, we explored the potential role of memory in the choice effects. For the choices between approach and avoidance items, we computed participants’ memory of the approach vs. avoidance status for each pair, and added memory as an extra predictor into the pre-registered analysis above. Similarly, for the choices between go and no-go items, we computed their memory of the go vs. no-go status for each pair, and added memory as a predictor in the pre-registered analysis. Lastly, we also explored whether the training led to changes in rating (as observed previously in Chen & Van Dessel, 2024), by computing the average change in ratings from before to after the training for each condition. The change scores were then analyzed with 2 (response, go vs. no-go) by 2 (consequence, approach vs. avoidance) Bayesian repeated-measures ANOVAs, for the two groups separately. For brevity, we mentioned the main findings from these exploratory analyses in the *Further exploratory analyses (not pre-registered)* subsection at the end of the Results section. More detailed information on the analyses and results are in the online Supplemental Materials. As a robustness check, for the exploratory analyses that involved data aggregation (i.e., on accuracy and response times in the training, scores in the memory tasks, and ratings before and after the training), we also conducted mixed-effects analysis, by using the maximal random effects structure on both the participant and the item level. The conclusions remained the same. Detailed results of these mixed-effects analyses are available in the analysis file on OSF.

#### Statistical inference

Default priors in *brms* were used. For each *brms* model, we ran 8 parallel chains, with 2000 iterations during the warm-up phase and 4000 iterations during the sampling phase. Inspection of the trace plots, the R-hat values, and the effective sample sizes showed that all models had converged and the estimates were stable. To make statistical inference, we used the equal-tailed percentile-based 95% credible intervals of the posterior distributions of parameter estimates. We deemed an effect statistically credible if the 95% CI excluded 0.

## Results

### Ratings before the training (pre-registered and not pre-registered)

For the items assigned into the Go-Approach vs. Go-Avoidance and the NoGo-Approach vs. NoGo-Avoidance choice trials, the average ratings between the approach and avoidance conditions were closely matched descriptively, for both when the response was go and no-go, and in both instruction groups (see Table 1).

**Table 1:** Ratings before the training for items used in the Go-Approach vs. Go-Avoidance and the NoGo-Approach vs. NoGo-Avoidance choice trials.


INSTRUCTION GROUP	RESPONSE	CONSEQUENCE	MEAN	SD

Approach/Avoidance	Go	Approach	18.82	48.27

		Avoidance	18.88	48.17

	NoGo	Approach	21.20	47.20

		Avoidance	20.82	47.13

Go/No-Go	Go	Approach	13.58	50.94

		Avoidance	13.82	51.04

	NoGo	Approach	19.12	50.45

		Avoidance	19.34	50.70


We pre-registered to conduct a 2 (response, go vs. no-go; within-subjects) by 2 (consequence, approach vs. avoidance; within-subjects) by 2 (instruction group, go/no-go vs. approach/avoidance; between-subjects) Bayesian repeated-measures ANOVA in JASP. By default, JASP (from version 0.16.3 onward, see Van Den Bergh et al., 2023) includes random slopes for repeated measures. However, in our particular case, this model specification did not work. Despite manually increasing the number of samples to 100000, the error percentages associated with the Bayes factors were still rather high (i.e., above 20%, see Van Den Bergh et al., 2023), suggesting that the estimates for Bayes factors were unstable. Indeed, in repeated runs of the same analysis, the Bayes factor estimates showed large variability, lending support for qualitatively different conclusions each time (for a demonstration of a similar issue, see Pfister, 2021). This issue emerged here, because the experimental program assigned the items into the approach and avoidance conditions in a counterbalanced manner for each participant, thereby minimizing individual differences in the effect of consequence. We therefore removed random slopes in the model specification, by selecting the ‘Legacy results’ option in JASP. In line with our predictions and the descriptive results presented above, for both the main effect of consequence and the interaction effect between consequence and instruction group, the Bayes factors provided support for the null hypothesis (Table 3). To further verify this result, we also conducted a frequentist ANOVA. The main effect of consequence was not statistically significant, *F*(1, 194) = 0.653, *p* = .420, nor was the interaction between consequence and instruction group, *F*(1, 194) = 0.126, *p* = .723. Before the training, participants therefore appeared to have no preference between the approach and avoidance items on these choice trials based on their ratings.

Similarly, for the items assigned into the Go-Approach vs. NoGo-Approach and the Go-Avoidance vs. NoGo-Avoidance choice trials, descriptively, the average ratings between the go and no-go conditions were closely matched, for both when the consequence was approach and avoidance, and in both instruction groups (see Table 2). Models including random slopes again did not produce stable Bayes factor estimates. We therefore computed Bayes factors based on models without random slopes. In line with our predictions, the Bayes factors provided support for the null hypothesis for both the main effect of response and the interaction effect between response and instruction group (Table 3). In a frequentist ANOVA, the main effect of response was not statistically significant, *F*(1, 194) = 0.250, *p* = .618, and the interaction between response and instruction group was also not statistically significant, *F*(1, 194) = 1.089, *p* = .298. Hence, before the training, participants seemed to have no preference between the go and no-go items on these choice trials.

**Table 2:** Ratings before the training for items used in the Go-Approach vs. NoGo-Approach and the Go-Avoidance vs. NoGo-Avoidance choice trials.


INSTRUCTION GROUP	CONSEQUENCE	RESPONSE	MEAN	SD

Approach/Avoidance	Approach	Go	15.08	52.23

		NoGo	14.86	52.64

	Avoidance	Go	27.41	44.97

		NoGo	27.49	45.14

Go/No-Go	Approach	Go	24.42	50.04

		NoGo	24.82	49.56

	Avoidance	Go	18.23	49.64

		NoGo	18.23	49.59


**Table 3:** Bayesian repeated-measures ANOVAs on ratings before the training and accuracies in the training.


EFFECTS	*BF*_01_ – AAT	*BF*_01_ – GNG	*BF*_10_ – ACCURACY

Response	4.84	12.23	7.19 × 10^29^

Consequence	12.72	6.58	7.45 × 10^15^

Instruction group	5.57	6.33	4637.7

Response * Consequence	9.25	9.68	1.47 × 10^32^

Response * Instruction group	7.71	9.66	5117.4

Consequence * Instruction group	9.24	0.016	495.1

Response * Consequence * Instruction group	6.67	6.66	1912.1


*Note*: *BF*_01_ – AAT shows the results from the Bayesian ANOVA on ratings before the training for items used in the Go-Approach vs. Go-Avoidance and the NoGo-Approach vs. NoGo-Avoidance choice trials. *BF*_01_ – GNG shows the results from the Bayesian ANOVA on ratings before the training for items used the Go-Approach vs. NoGo-Approach and the Go-Avoidance vs. NoGo-Avoidance choice trials. *BF*_10_ – Accuracy shows the results from the Bayesian ANOVA on accuracies in the training. Note that *BF*_01_ – AAT and *BF*_01_ – GNG quantify the support for the null hypothesis over the alternative hypothesis, while *BF*_10_ – Accuracy quantifies the support for the alternative hypothesis over the null hypothesis.

### Performance in the training (not pre-registered)

**Figure 3:**
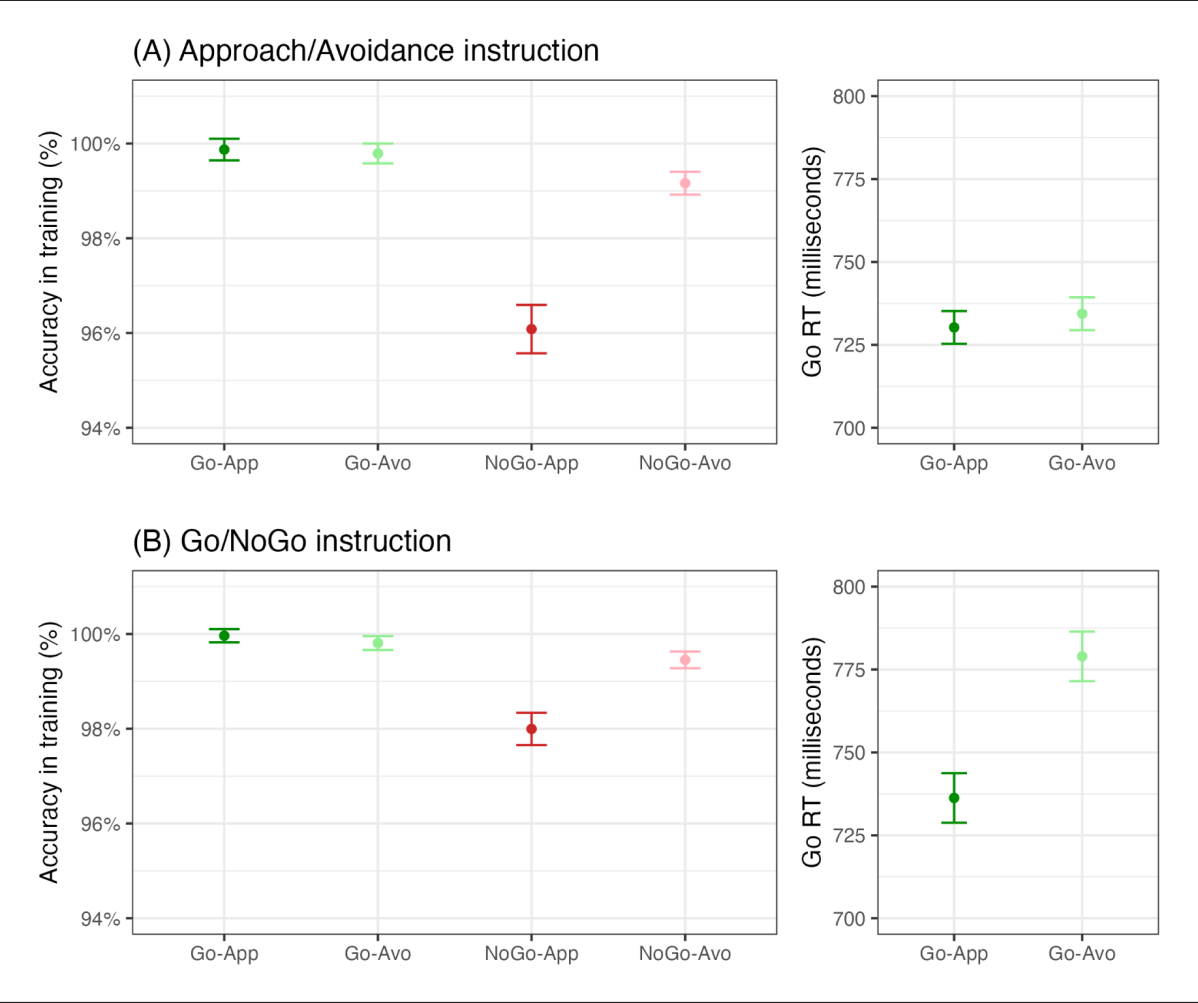
Performance in the training task in the **(A)** approach/avoidance and **(B)** go/no-go instruction group. The error bars stand for within-subject 95% confidence intervals.

The overall accuracy in the training was very high in both instruction groups (Figure 3). For the approach/avoidance instruction group, on the Go-Approach trials, *M* = 99.87%, *SD* = 0.92%; on the Go-Avoidance trials, *M* = 99.79%, *SD* = 0.58%; on the NoGo-Approach trials, *M* = 96.08%, *SD* = 3.03%; and on the NoGo-Avoidance trials, *M* = 99.16%, *SD* = 1.15%. For the go/no-go instruction group, on the Go-Approach trials, *M* = 99.96%, *SD* = 0.28%; on the Go-Avoidance trials, *M* = 99.81%, *SD* = 0.63%; on the NoGo-Approach trials, *M* = 98.00%, *SD* = 1.98%; and on the NoGo-Avoidance trials, *M* = 99.45%, *SD* = 0.80%.

Bayesian repeated-measures ANOVA (including random slopes) showed that for all main and interaction effects on accuracy there was extreme support for the alternative hypothesis (Table 3). To break down the three-way interaction effect, we conducted two separate Bayesian ANOVAs. First, we analyzed the accuracies on the go trials with a 2 (consequence, approach vs. avoidance) by 2 (instruction group, go/no-go vs. approach/avoidance) ANOVA. The Bayes factor provided moderate support for the main effect of consequence (*BF*_10_ =6.94), indicating that overall participants were more accurate when making go responses to approach than to avoid candies (although the difference appeared to be rather small). For both the main effect of instruction group and the interaction effect between consequence and instruction group, the Bayes factors provided moderate support for the null hypothesis (*BF*_01_ = 3.75 and 4.53, respectively). In a second analysis, we analyzed the accuracies on the no-go trials, similarly with a 2 (consequence, approach vs. avoidance) by 2 (instruction group, go/no-go vs. approach/avoidance) ANOVA. There was extreme support for the alternative hypothesis for the main effect of consequence (*BF*_10_ = 3.42 × 10^25^), the main effect of instruction group (*BF*_10_ = 1.71 × 10^5^), and their interaction effect (*BF*_10_ = 2041.4). Participants were more accurate when making no-go responses to avoid than to approach candies, and the effect of consequence on no-go accuracies was larger in the approach/avoidance group than in the go/no-go group.

Bayesian ANOVA on go response times showed support for the main effect of consequence (*BF*_10_ = 4.45 × 10^7^), the main effect of instruction group (*BF*_10_=5.48), and the interaction effect (*BF*_10_ = 1.17 × 10^6^). To break down the interaction effect, we conducted two paired-samples *t* tests, to compare the response times between Go-Approach and Go-Avoidance trials in the two instruction groups separately. The go/no-go group responded more quickly on the Go-Approach trials (*M* = 736.3 ms, *SD* = 69.2) than on the Go-Avoidance trials (*M* = 779.0 ms, *SD* = 91.7), diff = –42.7, *BF*_10_ = 3.20 × 10^9^, Cohen’s *d_z_* = 0.809. In contrast, for the approach/avoidance group, the mean response times did not differ between the Go-Approach trials (*M* = 730.3 ms, *SD* = 56.0) and the Go-Avoidance trials (*M* = 734.4 ms, *SD* = 55.1), diff = –4.1, *BF*_01_ = 4.61, Cohen’s *d_z_* = 0.118.

Approach consequences thus facilitated go responses, while avoidance consequences facilitated no-go responses in the training. This overall pattern of results was highly similar to the pattern observed in Chen and Van Dessel (2024).

### Choices between highly- and lowly-rated items (pre-registered)

Next, we examined participants’ choices on the filler trials, where they chose between a highly-rated and a lowly-rated item. As expected, the intercept (on the log odds ratio scale) was credibly larger than 0, estimate = 4.759, 95% CI = [4.348, 5.237], suggesting that participants overall chose highly-rated items more frequently than lowly-rated items. Furthermore, the estimate for the difference between the two instruction groups was not credible, estimate = 0.148, 95% CI = [–0.563, 0.859]. Both groups thus similarly preferred highly-rated items over lowly-rated items (for the go/no-go group, the probability of choosing highly-rated items was *M* = 97.96%, *SD* = 4.19%; for the approach/avoidance group, *M* = 97.58%, *SD* = 5.03%).

### Choices between approach and avoidance items (pre-registered)

**Figure 4:**
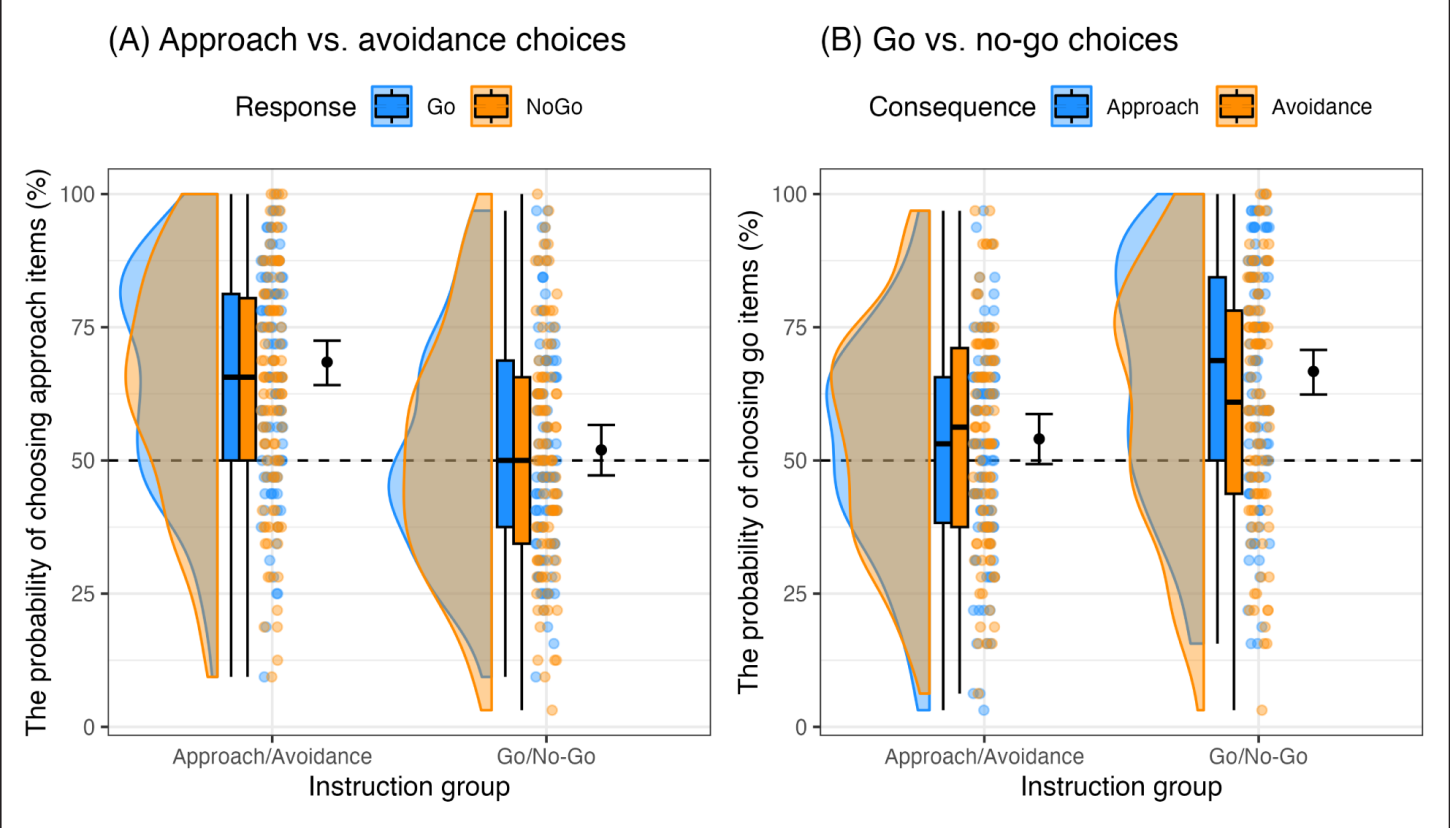
Choices between **(A)** approach and avoidance items and **(B)** go and no-go items. The blue and orange dots show for each participant, the probabilities of choosing approach items in **(A)**, and the probabilities of choosing go items in **(B)**. The black dots show for each group, the estimated probability of choosing approach items in **(A)**, and the estimated probability of choosing go items in **(B)**. The error bars stand for 95% credible intervals.

As shown above, before the training, participants had no preference between approach and avoidance items on the Go-Approach vs. Go-Avoidance and the NoGo-Approach and NoGo-Avoidance choice trials (Table 1). After the training, however, they overall chose approach items more frequently than avoidance items for consumption, intercept = 0.427, 95% CI = [0.288, 0.564]. In line with our prediction, the effect for instruction group was statistically credible, estimate = 0.696, 95% CI = [0.430, 0.968]. The approach/avoidance group chose approach items more than half of the time, estimated intercept on the log odds ratio scale = 0.775, 95% CI = [0.582, 0.969] (in probabilities, estimate = 68.5%, 95% CI = [64.1%, 72.5%]). In the go/no-go group, the estimated intercept did not differ credibly from 0, estimate = 0.079, 95% CI = [–0.112, 0.268] (in probabilities, estimate = 52.0%, 95% CI = [47.2%, 56.7%]; see Figure 4, panel A). The effect of response (go vs. no-go) and the interaction effect between response and instruction group were not credible, estimate = 0.114, 95% CI = [–0.154, 0.387], and estimate = –0.083, 95% CI = [–0.571, 0.410], respectively.

### Choices between go and no-go items (pre-registered)

Similarly, we found that before the training, participants had no preference between go and no-go items on the Go-Approach vs. NoGo-Approach and the Go-Avoidance vs. NoGo-Avoidance choice trials (Table 2). After the training, participants overall chose go items more frequently than no-go items for consumption, intercept = 0.429, 95% CI = [0.300, 0.556]. Importantly, the effect of instruction group was statistically credible, estimate = 0.533, 95% CI = [0.256, 0.814]. Participants in the go/no-go group preferred go over no-go items, estimated intercept = 0.695, 95% CI = [0.505, 0.883] (in probabilities, estimate = 66.7%, 95% CI = [62.4%, 70.7%]). In the approach/avoidance group, the preference for go items did not differ credibly from the chance level, estimate = 0.162, 95% CI = [–0.027, 0.352] (in probabilities, estimate = 54.0%, 95% CI = [49.3%, 58.7%]; see Figure 4, panel B). The effect of consequence (approach vs. avoidance) and the interaction effect between consequence and instruction group were not statistically credible, estimate = 0.062, 95% CI = [–0.171, 0.299], and estimate = 0.451, 95% CI = [–0.009, 0.915], respectively.

### Further exploratory analyses (not pre-registered)

We conducted some further exploratory analyses on performance in the memory tasks and ratings after the memory tasks. Briefly, the approach/avoidance group remembered the approach vs. avoidance conditions of items better than the go/no-go group, whereas the go/no-go group remembered the go vs. no-go conditions of items better than the approach/avoidance group. Participants’ memories of stimulus-action contingencies positively correlated with the effects of approach/avoidance and go/no-go actions on choices. More concretely, for the choices between approach and avoidance items, participants showed a stronger preference for approach over avoidance items when they had better memories of the approach vs. avoidance status of the items. Similarly, for the choices between go and no-go items, they showed a stronger preference for go over no-go items when they had better memories of the go vs. no-go status of the items. In both sets of analyses, the effects of instruction group were no longer statistically credible after including memory as an extra predictor. These results were consistent with previous findings with the go/no-go training (Chen & Veling, 2022) and the approach/avoidance training (Van Dessel, De Houwer, & Gast, 2016), which might suggest common cognitive mechanisms (e.g., forming propositions based on the learned contingencies) for these effects.

For ratings after the memory tasks, replicating Chen and Van Dessel (2024), we observed that for the approach/avoidance group, the ratings for avoided items decreased more than approached items, while there was no effect of go/no-go actions. In contrast, for the go/no-go group, the ratings for no-go items decreased more than go items, while there was no effect of approach/avoidance actions. Interested readers can find more detailed results from these analyses in the Supplemental Materials (https://osf.io/b7cka).

## Discussion

In the current research, we used a recently developed novel training that combined go/no-go and approach/avoidance actions in an orthogonal manner (Chen & Van Dessel, 2024), to examine the roles of action execution and action interpretation in determining how these actions influence food choices. In line with our predictions, when the instructions framed the responses as approach and avoidance actions, participants preferred approach items over avoidance items, but did not show a preference between go and no-go items in their choices. In contrast, when the instructions framed the responses as go and no-go actions, participants preferred go items over no-go items, but did not show a preference between approach and avoidance items. Action interpretation therefore determined whether go/no-go or approach/avoidance actions influenced food choice, despite that both groups of participants made the same motor responses in the training.

### Theoretical implications

The current results cannot be easily explained by the theoretical accounts that relate effects solely to the mere execution of go/no-go or approach/avoidance actions (see the Introduction). For go/no-go or approach/avoidance actions to influence stimulus evaluation (Chen & Van Dessel, 2024) and consumption choice (here), motor responses need to be *interpreted* as go/no-go or approach/avoidance actions. Furthermore, this interpretation of motor responses as certain actions is malleable, as the same responses can be framed as either go/no-go or approach/avoidance actions depending on the instructions and task cues, which determines the effects. This latter observation casts doubt on the claim that there are ‘hardwired’ links between specific go/approach responses and the appetitive system, and between specific no-go/avoidance responses and the aversive system, as proposed by some theoretical accounts (e.g., the devaluation-by-inhibition account for go/no-go actions, Guitart-Masip et al., 2014; Verbruggen et al., 2014; and the motivational systems account for approach/avoidance actions, Neumann et al., 2003).

Instead, the current results are in line with the theoretical accounts that emphasize action interpretation, namely the common coding account (Eder & Klauer, 2009; Eder & Rothermund, 2008) and the inferential account (Van Dessel, Hughes, & De Houwer, 2018b). According to these accounts, certain motor responses acquire valence, because they are interpreted as valenced actions. Although we did not test it directly here, we speculate that go/no-go and approach/avoidance actions acquired valence in the current context, because they were further interpreted as choosing versus not choosing something, which has clear evaluative meanings. That is, in the training, we operationalized approach as candies falling into one’s shopping cart, and avoidance as candies falling outside one’s shopping cart. Putting something inside or outside one’s shopping cart are presumably actions that people often use to choose wanted items or not choose unwanted items, such as when doing groceries. This can be one interpretation that leads approach actions to be perceived as positive and avoidance actions as negative in the current context. Similarly, people may interpret responding to something (i.e., making go responses) as selecting it, and not responding to something (i.e., making no-go responses) as not selecting it, which gives these actions evaluative meanings. This idea is in line with the findings by Houben (2023), who found that when go action was framed as ‘taking something’ and no-go action as ‘not taking something’, participants evaluated no-go items less positively than go items, as typically observed with the go/no-go training (Yang et al., 2022). However, when go action was framed as ‘throwing away something’ and no-go action as ‘keeping something’, the effect was reversed: participants evaluated no-go items *more* positively than go items. Interpreting go/approach actions as choosing something, and no-go/avoidance actions as not choosing something may be one common mental process that drives the effects of both types of actions in the current study. This idea further suggests that one might also be able to reverse approach-avoidance effects. Specifically, approach actions (i.e., decreasing the distance between an object and oneself) are not necessarily interpreted as positive, and avoidance actions (i.e., increasing the distance between an object and oneself) are also not necessarily interpreted as negative. Rather, the specific interpretations of these actions will strongly depend on the context. This can be tested with the current training paradigm. For instance, instead of using a shopping cart, we may use an image of a trash bin in the training. In such a context, approach means discarding something, whereas avoidance means not discarding something (similar to how the meanings of go and no-go actions were reversed in Houben, 2023). We expect that after such a training, participants will evaluate avoidance items more positively than approach items, which will further support the idea that context-dependent action interpretation underlies the valence that we ascribe to certain motor responses.

As we speculated above, the effects of go/no-go and approach/avoidance actions may, in the current study, originate from interpreting these actions as choosing versus not choosing something. In this case, the go/no-go training and the approach/avoidance training essentially amount to training people to practice choosing certain items and not choosing other items (for related ideas on the go/no-go training, see Veling et al., 2022). Practicing such choices may then influence subsequent stimulus evaluation and consumption choice. This perspective raises novel theoretical questions on how such motor response trainings may be related to other lines of research. One prominent phenomenon of potential relevance is choice-induced preference change, where the act of choosing between equally desirable options increases people’s preference for chosen over unchosen options after making a choice (Brehm, 1956; Enisman et al., 2021). Interestingly, choice-induced preference change can emerge even without people actually making a choice. Rather, having a *belief* about making the choice seems sufficient to bring about the effect (Enisman et al., 2021). This observation dovetails with our theoretical proposition that action interpretation may be more fundamental than action execution in determining how actions influence evaluation and choice.

Choices can be expressed without making any overt motor responses (e.g., deciding to not eat a piece of chocolate in one’s mind). This raises the question of whether action execution is necessary for the effects of go/no-go and approach/avoidance actions to emerge, if such actions indeed indicate choices. The answer to this question is currently mixed. Some previous work has shown that merely instructing participants to approach certain stimuli and avoid other stimuli was sufficient to change their evaluations of these stimuli, without them actually executing any responses (Smith et al., 2020; Van Dessel, Eder, & Hughes, 2018; Van Dessel et al., 2015). However, these instruction-based effects emerge primarily for unfamiliar, neutral stimuli, but not for valenced stimuli (Van Dessel et al., 2015, 2020, 2025). Related, merely observing a go/no-go training with highly appetitive foods was not sufficient to change people’s evaluation of these foods (Chen et al., 2016). Executing motor responses may thus still be necessary (or at least strengthens the effects), when using go/no-go and approach/avoidance actions to change the evaluations of valenced stimuli. Regardless of whether responses are overtly executed or covertly imagined (see e.g. Larsen et al., 2024; Moritz et al., 2019), interpreting these responses as certain valenced actions may be a prerequisite for these (executed or imagined) actions to bring about behavior change.

### Practical implications

The current results corroborate previous findings on stimulus evaluation (Chen & Van Dessel, 2024; Houben, 2023; Laham et al., 2014; Van Dessel, Eder, & Hughes, 2018), but also go beyond them, by showing the effect of action interpretation on consequential consumption choices. These findings have practical implications. People make numerous consumption decisions every day, and these decisions can lead to large cumulative effects on their health. To maximize the effects of motor response trainings in applied settings, the meaning or valence of trained motor responses should be sufficiently clear, allowing for congruent action interpretation. Action interpretation can be disambiguated via task instructions (Houben, 2023), and can be further strengthened by providing consequences after certain actions in the training (e.g., positive consequences after choosing healthy foods, and negative consequences after choosing unhealthy foods; Van Dessel, Hughes, & De Houwer, 2018a). Here we focused on go/no-go and approach/avoidance responses as rudimentary forms of actions. Actions in real life, however, are often much more elaborate than simple key presses or catching food items with a virtual shopping cart. These elaborate actions may in turn have clearer evaluative meanings in certain contexts, and incorporating these actions into trainings may further increase the effectiveness of these interventions (Larsen et al., 2024). These ideas need to be further examined in future research.

### Limitations and future directions

We note several limitations of the current study. First, we speculated above that go and approach actions were positive, because they were interpreted as choosing something, whereas no-go and avoidance actions were negative, because they were interpreted as not choosing something. However, the specific interpretations of these actions as choosing or not choosing something were neither measured nor manipulated here (c.f. Houben, 2023). To directly test this idea, future research can further manipulate or measure the specific meanings of go/no-go and approach/avoidance actions within the current training paradigm. Second, we used only candies in the current study. In applied settings, motor response trainings are often employed to promote the consumption of healthy foods and reduce the consumption of unhealthy foods. To test whether our training paradigm is effective in promoting healthier choices, healthy items can be consistently paired with go or approach actions, and unhealthy items with no-go or avoidance actions. Consistently pairing one type of action with one category of foods may also make the actions more meaningful (e.g., choose healthy, not choose unhealthy), which may make the training effects more long-lasting (Serfas et al., 2017). Lastly, although making choices for real consumption may be one step closer to real eating behavior than stimulus evaluation, the binary choices used here are still very far away from complex consumption behaviors in the real world. One important direction for future research is therefore to examine the role of action interpretation using more consequential behavioral outcomes, such as body weight (e.g., Lawrence et al., 2015; Veling et al., 2014).

## Conclusion

Framing the same motor responses as either go/no-go or approach/avoidance actions determined which action dimension influenced people’s consumption choice. This suggests that go/no-go and approach/avoidance responses are not inherently imbued with positive or negative valence. Instead, they may acquire and transfer valence via being interpreted as certain actions with evaluative meanings (e.g., as choosing and not choosing something). To maximize the effectiveness of motor response trainings, the trained responses therefore need to be framed in a way to facilitate their interpretation as clearly valenced and meaningful actions.

## Data Accessibility Statement

All experimental materials, raw data and analysis code are available at https://osf.io/24apk/.

## Footnotes

Note that most studies reviewed in Chen and Veling (2022) used a food choice task with a time limit (i.e., choosing within 1.5 seconds), while others did not implement such a time limit for making choices. Previous work has shown that the effect of GNG on choices was stronger when participants made choices more quickly (Chen et al., 2021, 2019). Since in the current study we used a food choice task with time limit, to compute the effect size of GNG we only included studies that have implemented a time limit in the choice task (486 participants in total).

## Appendix

**Table A1:** Study design table.

QUESTION	HYPOTHESIS	SAMPLING PLAN	ANALYSIS PLAN	RATIONALE FOR DECIDING THE SENSITIVITY OF THE TEST FOR CONFIRMING OR DISCONFIRMING THE HYPOTHESIS	INTERPRETATION GIVEN DIFFERENT OUTCOMES	THEORY THAT COULD BE SHOWN WRONG BY THE OUTCOMES

Does the effect of approach/avoidance actions on choice depend on action interpretation?	Approach/avoidance actions will more strongly influence choice when task instructions indicate approach/avoidance actions than when they indicate go/no-go actions. Furthermore, the approach/avoidance instruction group overall will choose approach items more frequently than avoidance items.	We will recruit 100 participants who are at least 18 years old in each group, resulting in 200 participants in total before any data exclusion. In case the sample size in an instruction group is below 90 after exclusions, we will continue recruiting participants for that specific instruction group, until the final sample size is 90.	Hierarchical logistic regression on choices on the Go-Approach vs.Go-Avoidance and NoGo-Approach vs. NoGo-Avoidance trials. Whether participants choose the Approach or Avoidance item is the dependent variable (Approach = 1, Avoidance = 0). Whether both items are Go or NoGo is used as a within-subjects predictor, and the instruction group is used as a between-subjects predictor. Random intercept and random slope per participant, left candy and right candy are included.	Previous work found an effect size of Cohen’s d of 0.533 for GNG on food choices. We expect the effect of AAT on food choices to be at least as big as that of GNG. To be conservative, we use 80% of the expected effect size, which is 0.426. 60 participants are needed (with a two-sided one sample t test) for 90% power with an alpha level of .05. Furthermore, instruction increased the effect of actions on stimulus evaluation in previous work, with Hedge’s g = 0.611 and 0.923 for GNG and AAT, respectively. Again, using 80% of 0.611 (0.488), 90 participants per condition (180 in total) are needed (with a two-sided independent samples t test) for 90% power with an alpha level of .05. To leave room for potential exclusions, we will initially recruit 100 participants per group (200 in total).	If task cues influence the effect of AAT on choice, this would indicate that the effect of approach/avoidance actions on choice depends on action interpretation. If not, this would indicate that different from stimulus evaluation, choice change by AAT is not dependent on action interpretation.	The results will help us arbitrate between theoretical accounts that emphasize action execution in the effects by go/no-go and approach/avoi dance actions on choice, and those that emphasize action interpretation in the effects by go/no-go and approach/avoi dance actions.
		
Does the effect of go/no-go actions on choice depend on action interpretation?	Go/no-go actions will more strongly influence choice when task instructions indicate go/no-go actions than when they indicate approach/avoidance actions. Furthermore, the go/no-go instruction group overall will choose go items more frequently than no-go items.		Hierarchical logistic regression on choices on the Go-Approach vs. NoGo-Approach and Go-Avoidance vs. NoGo-Avoidance trials. Whether participants choose the Go or NoGo item is the dependent variable (Go = 1, NoGo = 0). Whether both items are Approach or Avoidance is used as a within-subjects predictor, and the instruction group is used as a between-subjects predictor. Random intercept and random slope per participant, left candy and right candy are included.		If task cues influence the effect of GNG on choice, this would indicate that the effect of go/no-go actions on choice depends on action interpretation. If not, this would indicate that different from stimulus evaluation, choice change by GNG is not dependent on action interpretation.	

## References

[1] Adams, R. C., Button, K. S., Hickey, L., Morrison, S., Smith, A., Bolus, W., Coombs, E., Randolph, S., Hunt, R., Kim, D., Chambers, C. D., & Lawrence, N. S. (2021). Food-related inhibitory control training reduces food liking but not snacking frequency or weight in a large healthy adult sample. Appetite, 167, 105601. 10.1016/j.appet.2021.105601 34284065

[2] Allom, V., & Mullan, B. (2015). Two inhibitory control training interventions designed to improve eating behaviour and determine mechanisms of change. Appetite, 89, 282–290. 10.1016/j.appet.2015.02.022 25725487

[3] Armel, K. C., Beaumel, A., & Rangel, A. (2008). Biasing simple choices by manipulating relative visual attention. Judgment and Decision Making, 3(5), 396–403. 10.1017/S1930297500000413

[4] Aulbach, M. B., Knittle, K., & Haukkala, A. (2019). Implicit process interventions in eating behaviour: A meta-analysis examining mediators and moderators. Health Psychology Review, 13(2), 179–208. 10.1080/17437199.2019.1571933 30676235

[5] Barr, D. J., Levy, R., Scheepers, C., & Tily, H. J. (2013). Random effects structure for confirmatory hypothesis testing: Keep it maximal. Journal of Memory and Language, 68(3), 255–278. 10.1016/j.jml.2012.11.001 PMC3881361 24403724

[6] Becker, D., Jostmann, N. B., Wiers, R. W., & Holland, R. W. (2015). Approach avoidance training in the eating domain: Testing the effectiveness across three single session studies. Appetite, 85, 58–65. 10.1016/j.appet.2014.11.017 25447011

[7] Brehm, J. W. (1956). Postdecision changes in the desirability of alternatives. The Journal of Abnormal and Social Psychology, 52(3), 384–389. 10.1037/h0041006 13318848

[8] Breslin, P. A. (2013). An Evolutionary Perspective on Food and Human Taste. Current Biology, 23(9), R409–R418. 10.1016/j.cub.2013.04.010 23660364 PMC3680351

[9] Bürkner, P.-C. (2022). Brms: Bayesian regression models using stan [R package version 2.18.0]. https://CRAN.R-project.org/package=brms

[10] Carbine, K. A., Muir, A. M., Allen, W. D., LeCheminant, J. D., Baldwin, S. A., Jensen, C. D., Kirwan, C. B., & Larson, M. J. (2021). Does inhibitory control training reduce weight and caloric intake in adults with overweight and obesity? A pre-registered, randomized controlled event-related potential (ERP) study. Behaviour Research and Therapy, 136, 103784. 10.1016/j.brat.2020.103784 33316579

[11] Centerbar, D. B., & Clore, G. L. (2006). Do Approach-Avoidance Actions Create Attitudes? Psychological Science, 17(1), 22–29. 10.1111/j.1467-9280.2005.01660.x 16371140

[12] Chen, Z., Holland, R., Quandt, J., Dijksterhuis, A., & Veling, H. (2021). How Preference Change Induced by Mere Action Versus Inaction Persists Over Time. Judgment and Decision Making, 16(1), 201–237. 10.31219/osf.io/b495y

[13] Chen, Z., Holland, R. W., Quandt, J., Dijksterhuis, A., & Veling, H. (2019). When mere action versus inaction leads to robust preference change. Journal of Personality and Social Psychology, 117(4), 721–740. 10.1037/pspa0000158 30920280

[14] Chen, Z., & Van Dessel, P. (2024). Action Interpretation Determines the Effects of Go/No-Go and Approach/Avoidance Actions on Stimulus Evaluation. Open Mind, 8, 898–923. 10.1162/opmi_a_00151 39077108 PMC11285421

[15] Chen, Z., & Veling, H. (2022). Toward a better understanding of durable behavior change by food Go/NoGo training. Current Opinion in Behavioral Sciences, 48, 101212. 10.1016/j.cobeha.2022.101212

[16] Chen, Z., Veling, H., Dijksterhuis, A., & Holland, R. W. (2016). How does not responding to appetitive stimuli cause devaluation: Evaluative conditioning or response inhibition? Journal of Experimental Psychology: General, 145(12), 1687–1701. 10.1037/xge0000236 27736134

[17] Chen, Z., Veling, H., Dijksterhuis, A., & Holland, R. W. (2018). Do impulsive individuals benefit more from food go/no-go training? Testing the role of inhibition capacity in the no-go devaluation effect. Appetite, 124, 99–110. 10.1016/j.appet.2017.04.024 28442335

[18] Chiu, Y.-C., & Egner, T. (2015a). Inhibition-Induced Forgetting Results from Resource Competition between Response Inhibition and Memory Encoding Processes. Journal of Neuroscience, 35(34), 11936–11945. 10.1523/JNEUROSCI.0519-15.2015 26311775 PMC4549404

[19] Chiu, Y.-C., & Egner, T. (2015b). Inhibition-Induced Forgetting: When More Control Leads to Less Memory. Psychological Science, 26(1), 27–38. 10.1177/0956797614553945 25398560 PMC4353579

[20] De Houwer, J., Crombez, G., Baeyens, F., & Hermans, D. (2001). On the generality of the affective Simon effect. Cognition & Emotion, 15(2), 189–206. 10.1080/02699930125883

[21] de Leeuw, J. R. (2015). jsPsych: A JavaScript library for creating behavioral experiments in a Web browser. Behavior Research Methods, 47(1), 1–12. 10.3758/s13428-014-0458-y 24683129

[22] Dignath, D., Eder, A. B., Steinhauser, M., & Kiesel, A. (2020). Conflict monitoring and the affective-signaling hypothesis—An integrative review. Psychonomic Bulletin & Review, 27(2), 193–216. 10.3758/s13423-019-01668-9 31898269

[23] Eder, A. B., & Klauer, K. C. (2009). A common-coding account of the bidirectional evaluation–behavior link. Journal of Experimental Psychology: General, 138(2), 218–235. 10.1037/a0015220 19397381

[24] Eder, A. B., & Rothermund, K. (2008). When do motor behaviors (mis)match affective stimuli? An evaluative coding view of approach and avoidance reactions. Journal of Experimental Psychology: General, 137(2), 262–281. 10.1037/0096-3445.137.2.262 18473659

[25] Enisman, M., Shpitzer, H., & Kleiman, T. (2021). Choice changes preferences, not merely reflects them: A meta-analysis of the artifact-free free-choice paradigm. Journal of Personality and Social Psychology, 120(1), 16–29. 10.1037/pspa0000263 33411557

[26] Faul, F., Erdfelder, E., Lang, A.-G., & Buchner, A. (2007). G*Power 3: A flexible statistical power analysis program for the social, behavioral, and biomedical sciences. Behavior Research Methods, 39(2), 175–191. 10.3758/BF03193146 17695343

[27] Forman, E. M., Manasse, S. M., Dallal, D. H., Crochiere, R. J., Loyka, C. M., Butryn, M. L., Juarascio, A. S., & Houben, K. (2019). Computerized neurocognitive training for improving dietary health and facilitating weight loss. Journal of Behavioral Medicine, 42(6), 1029–1040. 10.1007/s10865-019-00024-5 30891657 PMC6752994

[28] Gabry, J., & Češnovar, R. (2022). Cmdstanr: R interface to cmdstan [https://mc-stan.org/cmdstanr/].

[29] Gabry, J., & Mahr, T. (2022). Bayesplot: Plotting for bayesian models [R package version 1.10.0]. https://mc-stan.org/bayesplot/

[30] Guitart-Masip, M., Duzel, E., Dolan, R., & Dayan, P. (2014). Action versus valence in decision making. Trends in Cognitive Sciences, 18(4), 194–202. 10.1016/j.tics.2014.01.003 24581556 PMC3989998

[31] Herman, C. P., & Polivy, J. (1980). Restrained eating. In A. J. Stunkard (Ed.), Obesity (pp. 208–225). Saunders.

[32] Hope, R. M. (2022). Rmisc: Ryan miscellaneous [R package version 1.5.1]. https://CRAN.R-project.org/package=Rmisc

[33] Houben, K. (2023). How does Go/No-Go training lead to food devaluation? Separating the effects of motor inhibition and response valence. Cognition and Emotion, 1–14. 10.1080/02699931.2023.2208339 37144522

[34] Houben, K., & Aulbach, M. (2023). Is there a difference between stopping and avoiding? A review of the mechanisms underlying Go/No-Go and Approach-Avoidance training for food choice. Current Opinion in Behavioral Sciences, 49, 101245. 10.1016/j.cobeha.2022.101245

[35] Itzkovitch, A., Bar Or, M., & Schonberg, T. (2022). Cue-approach training for food behavior. Current Opinion in Behavioral Sciences, 47, 101202. 10.1016/j.cobeha.2022.101202

[36] JASP Team. (2024). JASP (Version 0.19.0)[Computer software]. https://jasp-stats.org/

[37] Johannes, N., Buijzen, M., & Veling, H. (2021). Beyond inhibitory control training: Inactions and actions influence smartphone app use through changes in explicit liking. Journal of Experimental Psychology: General, 150(3), 431–445. 10.1037/xge0000888 32881564

[38] Kassambara, A. (2023). Ggpubr: Ggplot2 based publication ready plots [R package version 0.6.0]. https://rpkgs.datanovia.com/ggpubr/

[39] Kawakami, K., Phills, C. E., Steele, J. R., & Dovidio, J. F. (2007). (Close) distance makes the heart grow fonder: Improving implicit racial attitudes and interracial interactions through approach behaviors. Journal of Personality and Social Psychology, 92(6), 957–971. 10.1037/0022-3514.92.6.957 17547482

[40] Kay, M. (2022). Tidybayes: Tidy data and geoms for bayesian models [R package version 3.0.2]. https://CRAN.R-project.org/package=tidybayes

[41] Laham, S. M., Kashima, Y., Dix, J., Wheeler, M., & Levis, B. (2014). Elaborated contextual framing is necessary for action-based attitude acquisition. Cognition and Emotion, 28(6), 1119–1126. 10.1080/02699931.2013.867833 24354687

[42] Larsen, J. K., Hollands, G. J., Moritz, S., Wiers, R. W., & Veling, H. (2024). How can imaginal retraining for modifying food craving be improved? Appetite, 202, 107639. 10.1016/j.appet.2024.107639 39163917

[43] Lawrence, N. S., O’Sullivan, J., Parslow, D., Javaid, M., Adams, R. C., Chambers, C. D., Kos, K., & Verbruggen, F. (2015). Training response inhibition to food is associated with weight loss and reduced energy intake. Appetite, 95, 17–28. 10.1016/j.appet.2015.06.009 26122756 PMC4596151

[44] Liu, H., Holland, R. W., & Veling, H. (2023). When not responding to food changes food value: The role of timing. Appetite, 106583. 10.1016/j.appet.2023.106583 37121485

[45] Lüdecke, D. (2022). Sjplot: Data visualization for statistics in social science [R package version 2.8.12]. https://strengejacke.github.io/sjPlot/

[46] Makowski, D., Lüdecke, D., Ben-Shachar, M. S., Patil, I., Wilson, M. D., & Wiernik, B. M. (2022). Bayestestr: Understand and describe bayesian models and posterior distributions [R package version 0.13.0]. https://easystats.github.io/bayestestR/

[47] Memarian, S., Moradi, A., Hasani, J., & Mullan, B. (2021). Can sweet food-specific inhibitory control training via a mobile application improve eating behavior in children with obesity? British Journal of Health Psychology, bjhp.12566. 10.1111/bjhp.12566 34676624

[48] Moritz, S., Paulus, A. M., Hottenrott, B., Weierstall, R., Gallinat, J., & Kühn, S. (2019). Imaginal retraining reduces alcohol craving in problem drinkers: A randomized controlled trial. Journal of Behavior Therapy and Experimental Psychiatry, 64, 158–166. 10.1016/j.jbtep.2019.04.001 31071483

[49] Najberg, H., Rigamonti, M., Mouthon, M., & Spierer, L. (2021). Modifying food items valuation and weight with gamified executive control training. Royal Society Open Science, 8(5), 191288. 10.1098/rsos.191288 34084536 PMC8150012

[50] Neumann, R., Förster, J., & Strack, F. (2003). Motor compatibility: The bidirectional link between behavior and evaluation. In J. Musch & K. C. Klauer (Eds.), The psychology of evaluation: Affective processes in cognition and emotion (pp. 371–391). Lawrence Erlbaum Associates. 10.4324/9781410606853-22

[51] Pfister, R. (2021). Variability of Bayes Factor estimates in Bayesian Analysis of Variance. The Quantitative Methods for Psychology, 17(1), 40–45. 10.20982/tqmp.17.1.p040

[52] Porter, L., Bailey-Jones, C., Priudokaite, G., Allen, S., Wood, K., Stiles, K., Parvin, O., Javaid, M., Verbruggen, F., & Lawrence, N. (2018). From cookies to carrots; the effect of inhibitory control training on children’s snack selections. Appetite, 124, 111–123. 10.1016/j.appet.2017.05.010 28479406

[53] Quandt, J., Holland, R. W., Chen, Z., & Veling, H. (2019). The role of attention in explaining the no-go devaluation effect: Effects on appetitive food items. Journal of Experimental Psychology: Human Perception and Performance, 45(8), 1119–1133. 10.1037/xhp0000659 31144856

[54] R Core Team. (2022). R: A language and environment for statistical computing. Vienna, Austria: R Foundation for Statistical Computing. https://www.R-project.org/

[55] Rinck, M., & Becker, E. S. (2007). Approach and avoidance in fear of spiders. Journal of Behavior Therapy and Experimental Psychiatry, 38(2), 105–120. 10.1016/j.jbtep.2006.10.001 17126289

[56] Ripley, B. (2022). Mass: Support functions and datasets for venables and ripley’s mass [R package version 7.3-58.1]. http://www.stats.ox.ac.uk/pub/MASS4/

[57] Schonberg, T., Bakkour, A., Hover, A. M., Mumford, J. A., Nagar, L., Perez, J., & Poldrack, R. A. (2014). Changing value through cued approach: An automatic mechanism of behavior change. Nature Neuroscience, 17(4), 625–630. 10.1038/nn.3673 24609465 PMC4041518

[58] Schonberg, T., & Katz, L. N. (2020). A Neural Pathway for Nonreinforced Preference Change. Trends in Cognitive Sciences, 24, 504–514. 10.1016/j.tics.2020.04.002 32430228

[59] Serfas, B. G., Florack, A., Büttner, O. B., & Voegeding, T. (2017). What does it take for sour grapes to remain sour? Persistent effects of behavioral inhibition in go/no-go tasks on the evaluation of appetitive stimuli. Motivation Science, 3(1), 1–18. 10.1037/mot0000051

[60] Singmann, H., Bolker, B., Westfall, J., Aust, F., & Ben-Shachar, M. S. (2022). Afex: Analysis of factorial experiments [R package version 1.2-0]. https://CRAN.R-project.org/package=afex

[61] Smith, C. T., Calanchini, J., Hughes, S., Van Dessel, P., & De Houwer, J. (2020). The impact of instruction- and experience-based evaluative learning on IAT performance: A Quad model perspective. Cognition and Emotion, 34(1), 21–41. 10.1080/02699931.2019.1592118 30898017

[62] Stice, E., Yokum, S., Veling, H., Kemps, E., & Lawrence, N. S. (2017). Pilot test of a novel food response and attention training treatment for obesity: Brain imaging data suggest actions shape valuation. Behaviour Research and Therapy, 94, 60–70. 10.1016/j.brat.2017.04.007 28505470 PMC5656010

[63] Tzavella, L., Lawrence, N. S., Button, K. S., Hart, E. A., Holmes, N. M., Houghton, K., Badkar, N., Macey, E., Braggins, A.-J., Murray, F. C., Chambers, C. D., & Adams, R. C. (2021). Effects of go/no-go training on food-related action tendencies, liking and choice. Royal Society Open Science, 8(8), 210666. 10.1098/rsos.210666 34457346 PMC8385366

[64] Van Den Bergh, D., Wagenmakers, E.-J., & Aust, F. (2023). Bayesian Repeated-Measures Analysis of Variance: An Updated Methodology Implemented in JASP. Advances in Methods and Practices in Psychological Science, 6(2), 25152459231168024. 10.1177/25152459231168024

[65] Van Dessel, P., De Houwer, J., & Gast, A. (2016). Approach–Avoidance Training Effects Are Moderated by Awareness of Stimulus–Action Contingencies. Personality and Social Psychology Bulletin, 42(1), 81–93. 10.1177/0146167215615335 26567171

[66] Van Dessel, P., De Houwer, J., Gast, A., Roets, A., & Smith, C. T. (2020). On the effectiveness of approach-avoidance instructions and training for changing evaluations of social groups. Journal of Personality and Social Psychology, 119(2), e1–e14. 10.1037/pspa0000189 32150429

[67] Van Dessel, P., De Houwer, J., Gast, A., & Smith, C. T. (2015). Instruction-Based Approach-Avoidance Effects: Changing Stimulus Evaluation via the Mere Instruction to Approach or Avoid Stimuli. Experimental Psychology, 62(3), 161–169. 10.1027/1618-3169/a000282 25516008

[68] Van Dessel, P., De Houwer, J., Roets, A., & Gast, A. (2016). Failures to change stimulus evaluations by means of subliminal approach and avoidance training. Journal of Personality and Social Psychology, 110(1), e1–e15. 10.1037/pspa0000039 26524002

[69] Van Dessel, P., Eder, A. B., & Hughes, S. (2018). Mechanisms underlying effects of approach-avoidance training on stimulus evaluation. Journal of Experimental Psychology: Learning, Memory, and Cognition, 44(8), 1224–1241. 10.1037/xlm0000514 29648864

[70] Van Dessel, P., Hughes, S., & De Houwer, J. (2018a). Consequence-Based Approach-Avoidance Training: A New and Improved Method for Changing Behavior. Psychological Science, 29(12), 1899–1910. 10.1177/0956797618796478 30312146

[71] Van Dessel, P., Hughes, S., & De Houwer, J. (2018b). How Do Actions Influence Attitudes? An Inferential Account of the Impact of Action Performance on Stimulus Evaluation. Personality and Social Psychology Review, 108886831879573. 10.1177/1088868318795730 30229697

[72] Van Dessel, P., Hughes, S., Perugini, M., Smith, C. T., Mao, Z.-F., & De Houwer, J. (2025). The role of awareness and demand in evaluative learning. Journal of Personality and Social Psychology. 10.1037/pspa0000423 39760742

[73] Vandenbosch, K., & De Houwer, J. (2011). Failures to induce implicit evaluations by means of approach–avoid training. Cognition and Emotion, 25(7), 1311–1330. 10.1080/02699931.2011.596819 21933034

[74] Vehtari, A., Gabry, J., Magnusson, M., Yao, Y., Bürkner, P.-C., Paananen, T., & Gelman, A. (2022). Loo: Efficient leave-one-out cross-validation and waic for bayesian models [R package version 2.5.1]. https://CRAN.R-project.org/package=loo

[75] Veling, H., Aarts, H., & Stroebe, W. (2013a). Stop signals decrease choices for palatable foods through decreased food evaluation. Frontiers in Psychology, 4. 10.3389/fpsyg.2013.00875 PMC3840792 24324451

[76] Veling, H., Aarts, H., & Stroebe, W. (2013b). Using stop signals to reduce impulsive choices for palatable unhealthy foods. British Journal of Health Psychology, 18(2), 354–368. 10.1111/j.2044-8287.2012.02092.x 23017096

[77] Veling, H., Becker, D., Liu, H., Quandt, J., & Holland, R. W. (2022). How go/no-go training changes behavior: A value-based decision-making perspective. Current Opinion in Behavioral Sciences, 47, 101206. 10.1016/j.cobeha.2022.101206

[78] Veling, H., Chen, Z., Tombrock, M. C., Verpaalen, I. A. M., Schmitz, L. I., Dijksterhuis, A., & Holland, R. W. (2017). Training impulsive choices for healthy and sustainable food. Journal of Experimental Psychology: Applied, 23(2), 204–215. 10.1037/xap0000112 28150960

[79] Veling, H., Holland, R. W., & van Knippenberg, A. (2008). When approach motivation and behavioral inhibition collide: Behavior regulation through stimulus devaluation. Journal of Experimental Social Psychology, 44(4), 1013–1019. 10.1016/j.jesp.2008.03.004

[80] Veling, H., Lawrence, N. S., Chen, Z., van Koningsbruggen, G. M., & Holland, R. W. (2017). What Is Trained During Food Go/No-Go Training? A Review Focusing on Mechanisms and a Research Agenda. Current Addiction Reports, 4(1), 35–41. 10.1007/s40429-017-0131-5 28357193 PMC5350201

[81] Veling, H., van Koningsbruggen, G. M., Aarts, H., & Stroebe, W. (2014). Targeting impulsive processes of eating behavior via the internet. Effects on body weight. Appetite, 78, 102–109. 10.1016/j.appet.2014.03.014 24675683

[82] Veling, H., Verpaalen, I. A., Liu, H., Mosannenzadeh, F., Becker, D., & Holland, R. W. (2021). How can food choice best be trained? Approach-avoidance versus go/no-go training. Appetite, 163, 105226. 10.1016/j.appet.2021.105226 33766617

[83] Verbruggen, F., Best, M., Bowditch, W. A., Stevens, T., & McLaren, I. P. (2014). The inhibitory control reflex. Neuropsychologia, 65, 263–278. 10.1016/j.neuropsychologia.2014.08.014 25149820

[84] Vermeylen, L., Wisniewski, D., González-García, C., Hoofs, V., Notebaert, W., & Braem, S. (2020). Shared Neural Representations of Cognitive Conflict and Negative Affect in the Medial Frontal Cortex. The Journal of Neuroscience, 40(45), 8715–8725. 10.1523/JNEUROSCI.1744-20.2020 33051353 PMC7643292

[85] Wagenmakers, E.-J., Love, J., Marsman, M., Jamil, T., Ly, A., Verhagen, J., Selker, R., Gronau, Q. F., Dropmann, D., Boutin, B., Meerhoff, F., Knight, P., Raj, A., van Kesteren, E.-J., van Doorn, J., Šmíra, M., Epskamp, S., Etz, A., Matzke, D., … Morey, R. D. (2018). Bayesian inference for psychology. Part II: Example applications with JASP. Psychonomic Bulletin & Review, 25(1), 58–76. 10.3758/s13423-017-1323-7 28685272 PMC5862926

[86] Wickham, H. (2022). Tidyverse: Easily install and load the tidyverse [R package version 1.3.2]. https://CRAN.R-project.org/package=tidyverse

[87] Woud, M. L., Maas, J., Becker, E. S., & Rinck, M. (2013). Make the manikin move: Symbolic approach–avoidance responses affect implicit and explicit face evaluations. Journal of Cognitive Psychology, 25(6), 738–744. 10.1080/20445911.2013.817413

[88] Wu, Q., Xia, H., Shields, G. S., Nie, H., Li, J., Chen, H., & Yang, Y. (2023). Neural correlates underlying preference changes induced by food Go/No-Go training. Appetite, 186, 106578. 10.1016/j.appet.2023.106578 37150052

[89] Xie, Y. (2022). Knitr: A general-purpose package for dynamic report generation in r [R package version 1.41]. https://yihui.org/knitr/

[90] Yang, Y., Morys, F., Wu, Q., Li, J., & Chen, H. (2021). Pilot study of food-specific go/no-go training for overweight individuals: Brain imaging data suggest inhibition shapes food evaluation. Social Cognitive and Affective Neuroscience, 18(1), nsab137. 10.1093/scan/nsab137 PMC10074770 34918164

[91] Yang, Y., Qi, L., Morys, F., Wu, Q., & Chen, H. (2022). Food-Specific Inhibition Training for Food Devaluation: A Meta-Analysis. Nutrients, 14(7), 1363. 10.3390/nu14071363 35405975 PMC9002952

[92] Yang, Y., Shields, G. S., Wu, Q., Liu, Y., Chen, H., & Guo, C. (2019). Cognitive training on eating behaviour and weight loss: A meta-analysis and systematic review. Obesity Reviews, 20(11), 1628–1641. 10.1111/obr.12916 31353774

[93] Zhu, H. (2021). Kableextra: Construct complex table with kable and pipe syntax [R package version 1.3.4]. https://CRAN.R-project.org/package=kableExtra

